# The role of physician apprenticeship models in the cost of caring: an AI-assisted systematic scoping review

**DOI:** 10.1186/s12909-026-09932-y

**Published:** 2026-07-27

**Authors:** Suzanne Pei Lin Goh, Wesley Teck Wee Loo, Nur Amira Binte Abdul Hamid, Priyadarshini A, Vengadasalam Murugam, Lalit Kumar Radha Krishna

**Affiliations:** 1 Duke-NUS Medical School, 8 College Road, Singapore, 169857 Singapore; 2 KK Women’s and Children’s Hospital, 100 Bukit Timah Road, Singapore, 229899 Singapore; 3 Division of Supportive and Palliative Care, National Cancer Centre Singapore, 30 Hospital Boulevard, Singapore, 168583 Singapore; 4 Division of Cancer Education, National Cancer Centre Singapore, 30 Hospital Boulevard, Singapore, 168583 Singapore; 5 SingHealth Duke-NUS Medical Humanities Institute, 8 College Road, Singapore, 169857 Singapore; 6 School of Health Sciences, University of Dundee, 11 Airlie Pl, Dundee, Dundee, DD1 4HJ UK; 7 Medical Affairs, National Cancer Centre Singapore, 30 Hospital Boulevard, Singapore, 168583 Singapore; 8 PalC, The Palliative Care Centre for Excellence in Research and Education, PalC c/o Dover Park Hospice, 10 Jalan Tan Tock Seng, Singapore, 308436 Singapore; 9 Palliative Care Institute Liverpool, Academic Palliative & End of Life Care Centre, University of Liverpool, 200 London Road, Liverpool, Liverpool, L3 9TA UK; 10 Health Data Science, University of Liverpool, Whelan Building, The Quadrangle, Brownlow Hill, Liverpool, Liverpool, L69 3GB UK; 11 Cynthia Goh Palliative Care Institute, National Cancer Centre Singapore, 30 Hospital Boulevard, Singapore, 168583 Singapore

**Keywords:** Apprenticeship, Mentoring, Physicians, Ttrainees, Cost of caring, Compassion fatigue, Moral distress, Vicarious trauma, Secondary traumatic stress, Burnout

## Abstract

**Background:**

Emotionally and psychologically demanding specialties place physicians at heightened risk of compassion fatigue, moral distress, vicarious trauma, secondary traumatic stress and burnout. Collectively, this cost of caring can impede professional identity formation, the developmental process through which individuals learn to think, act, and feel as physicians. Apprenticeship models, characterized by longitudinal mentored relationships, progressive entrustment, and guided socialization into communities of professional practice, offer a theoretically promising approach to addressing these burdens. However, systematic evidence on how apprenticeship models influence physicians experiencing the cost of caring remains limited. This systematic scoping review synthesizes evidence on apprenticeship characteristics, reported burdens and observed outcomes.

**Methods:**

This review was guided by the modified Systematic Evidence-Based Approach, which integrates artificial intelligence (AI) tools to supplement manual searching and analysis. Literature searches were conducted for publications between January 1, 2000 and December 30, 2025 on PubMed, Embase, ERIC, Scopus and Google Scholar databases, supplemented by Elicit AI. Of 7,334 abstracts identified, 481 full-text articles were reviewed, and 99 met the inclusion criteria. Human-led thematic and directed content analyses were performed to synthesize themes, with findings triangulated against outputs from ChatGPT 5 Pro.

**Results:**

Five themes were identified: socialization of apprentices, situated learning, critical reflection, impact on supervisors, and limitations of apprenticeship. Across included studies, apprenticeship features, including mentor-facilitated discussion of moral challenges, structured reflective practice, and sustained supervisory relationships, were associated with reduced moral distress, lower burnout, and preserved empathy. Applying Krishna and Alsuwaigh’s Ring Theory of Personhood and Krishna’s Internal Compass frameworks suggest that apprenticeship, when functioning well, may support physicians across multiple dimensions of personhood simultaneously, offering a developmental architecture that symptom-specific interventions do not reliably provide.

**Conclusion:**

Direct empirical evidence linking specific apprenticeship mechanisms to cost of caring outcomes remains limited, and the connections proposed in this review are largely theory-informed. The synthesis presented offers a theoretical foundation for intervention design and future empirical research. Future studies should employ longitudinal designs to examine whether strengthening the Internal Compass through apprenticeship features produces reductions in the cost of caring, with attention to cross-cultural applicability and the sustainability of the supervisory role.

**Trial registration:**

10.17605/OSF.IO/GHTA6

**Supplementary Information:**

The online version contains supplementary material available at https://doi.org/10.1186/s12909-026-09932-y.

## Background

Physicians in emotionally demanding specialties face psychological burdens collectively termed the “cost of caring” [1–5]. This encompasses compassion fatigue [1, 6], moral distress [3, 7], empathic strain [8, 9], vicarious trauma [1, 10], secondary traumatic stress [1, 11], and burnout [1, 12–14]. Particularly acute among clinicians caring for dying patients and distressed families, these burdens manifest as emotional exhaustion, erosion of empathy, and value conflicts that compromise wellbeing [2, 15–17]. Left unaddressed, they can precipitate attrition from emotionally demanding specialties and undermine patient care quality [18–22].

The cost of caring disrupts professional identity formation (PIF), the developmental process through which individuals learn to think, act and feel as physicians [23–25]. Repeated exposure to moral conflict and emotional exhaustion erodes the belief systems that anchor a physician’s sense of self, including their values, principles and professional norms [5, 26–28]. Students describe this as not feeling like they are “learning how to be a doctor” when formative experiences are characterized by distress rather than meaningful engagement [29].

Despite growing recognition of this crisis, many support mechanisms treat symptoms without addressing underlying identity disruption [30, 31]. Apprenticeship models, characterized by longitudinal mentorship, situated learning, and guided professional socialization, offer a theoretically promising approach to this deeper challenge. Their value lies in sustained, mentored engagement that embeds professional belief systems through repeated feedback, guided reflection, and meaning-making over time [32, 33]. Yet systematic evidence remains limited.

This systematic scoping review examines the characteristics, mechanisms, and outcomes of apprenticeship models among physicians experiencing the cost of caring. Findings are interpreted through two complementary frameworks developed by Krishna and colleagues, the Ring Theory of Personhood (RToP) [34–36] and the Internal Compass [27, 33], which together illuminate how apprenticeship may protect professional identity and mitigate the cost of caring.

### The Ring Theory of Personhood (RToP)

**Fig. 1:**
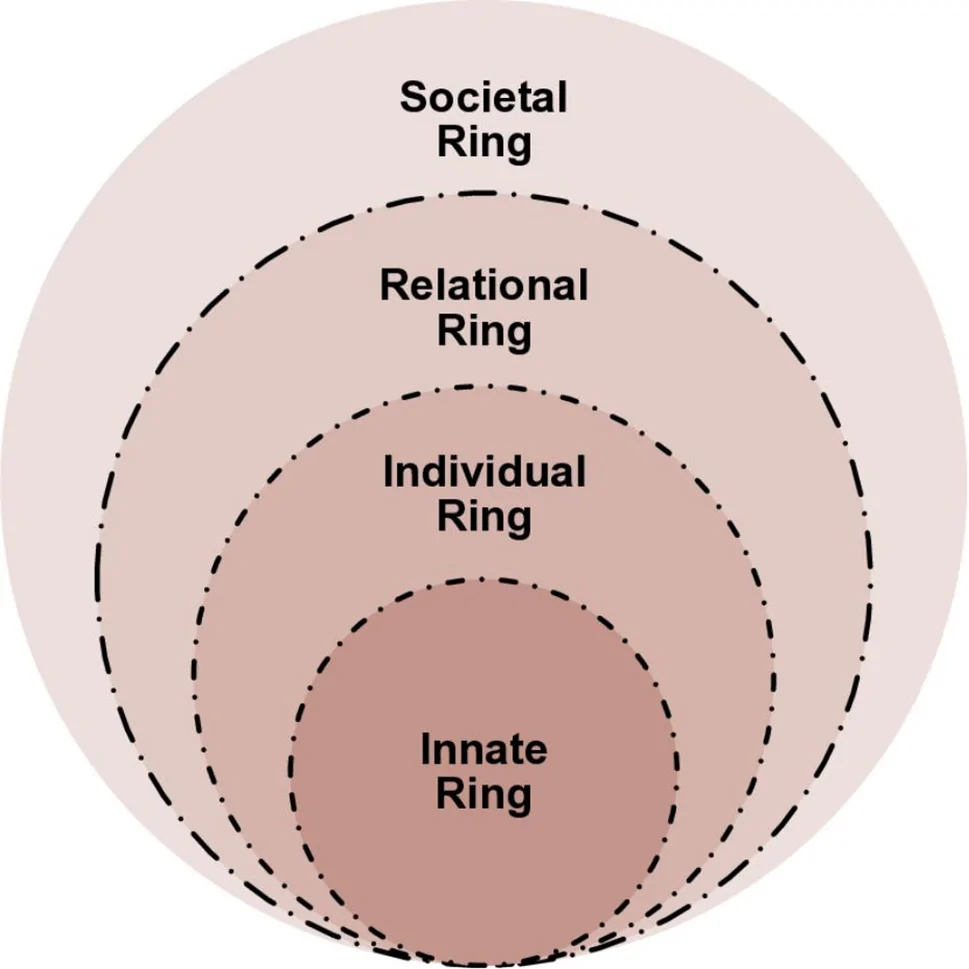
Ring theory of personhood. *Author-created figure*

The RToP conceptualizes personhood as comprising four nested rings [34, 35] (Fig. 1): the Innate Ring (core beliefs and sense of calling); the Individual Ring (emotions, personality traits, and cognitive schemas); the Relational Ring (relationships with family, friends, colleagues, and patients); and the Societal Ring (professional roles, cultural norms, legal frameworks, and ethical expectations). Disruptions to any ring through moral conflict, emotional exhaustion, or identity threat can accumulate and destabilize the physician’s broader sense of self.

### The internal compass

The Internal Compass describes an integrative psychological mechanism through which physicians navigate professional challenges [33]. It comprises five interrelated components: self-awareness, clinical judgment, ethical reasoning, professional confidence, and autonomous decision-making [33]. A well-developed Internal Compass enables physicians to reconcile competing demands, integrate difficult experiences, and maintain core professional values despite adversity [27, 33].

We propose that sustained mentored engagement supports the maturation of the Internal Compass, and that where this development is disrupted or incomplete, physicians may be more vulnerable to the cumulative burden of the cost of caring. Together, the longitudinal mentored relationships, progressive socialization, and structured reflective practice characteristic of apprenticeship may reinforce personhood across multiple rings of the RToP simultaneously, supporting professional roles and collegial relationships at the outer rings while protecting the core beliefs and sense of calling that the cost of caring most deeply threatens. This systematic scoping review examines the characteristics, mechanisms, and outcomes of apprenticeship models among physicians experiencing the cost of caring, interpreted through these two complementary frameworks.

## Methods

### Study design

**Fig. 2:**
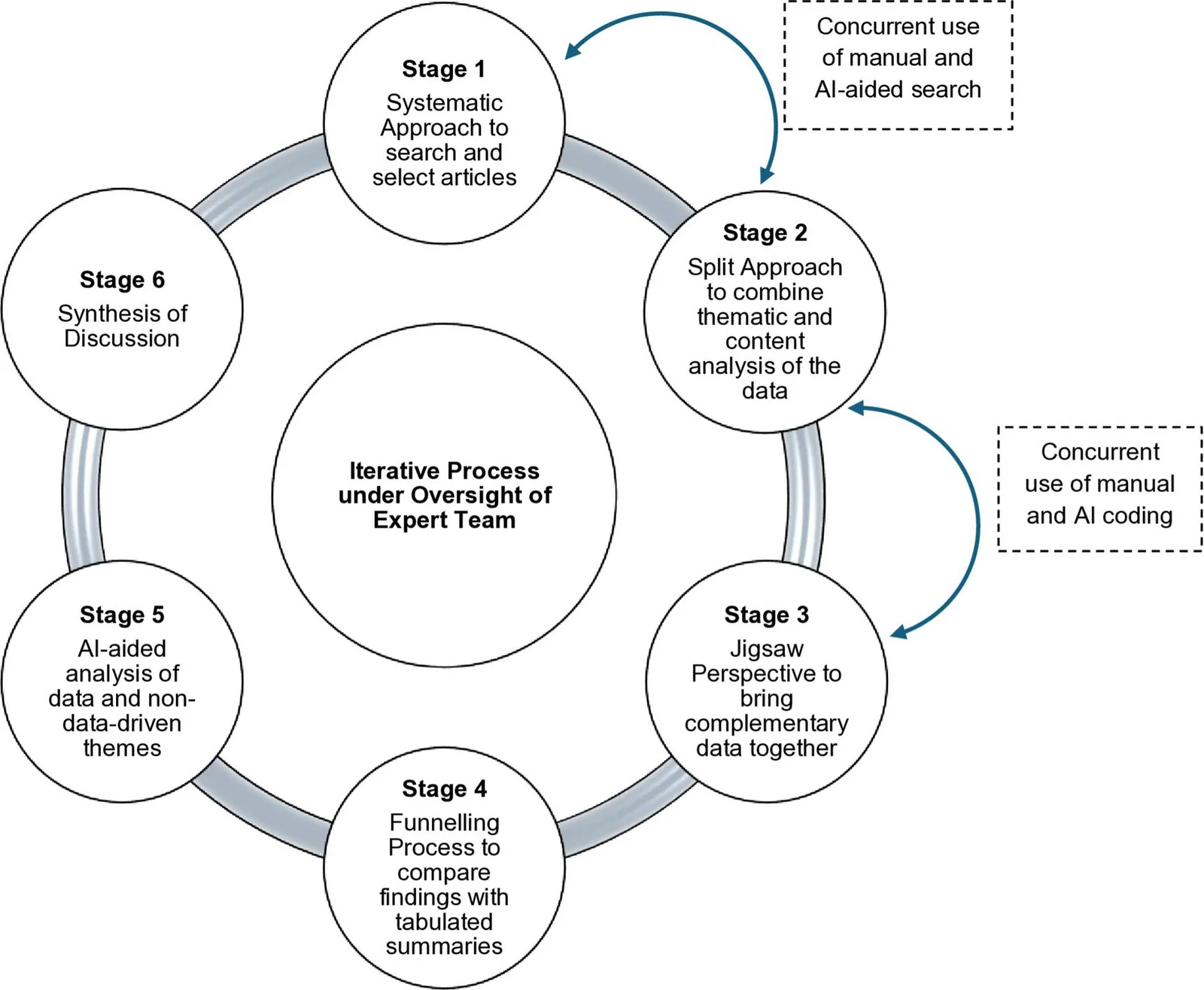
Stages of the modified systematic evidence-based approach. *Author-created figure*

This systematic scoping review followed the Preferred Reporting Items for Systematic Reviews and Meta-Analyses extension for Scoping Reviews (PRISMA-ScR) guidelines [37] (see Appendix S1). We employed a Modified Systematic Evidence-Based Approach (mSEBA), adapted from the Systematic Evidence-Based Approach previously used in systematic reviews of PIF and mentoring [33, 38–40]. The modified approach incorporated artificial intelligence (AI) tools to supplement manual searching and analysis [41]; all AI outputs were manually reviewed and validated. Figure 2 outlines the stages of mSEBA.

This study was retrospectively registered on the Open Science Framework on 15 June 2026 following formal registration protocols during revision. The study objectives, eligibility criteria, search strategy, and planned analyses as part of mSEBA were established a priori and were not modified following registration. The registration can be accessed at https://doi.org/10.17605/OSF.IO/GHTA6.

### Research question and eligibility criteria

The review asked: How do apprenticeship models affect physicians experiencing the cost of caring, and through what mechanisms?

Eligibility criteria followed the Population-Concept-Context (PCC) framework. We included studies examining apprenticeship models in medical education among physicians and trainees (junior physicians, residents, and medical students) in emotionally demanding specialties, including palliative care, oncology, emergency medicine, and surgery. Apprenticeship was operationally defined using established criteria in the health professions education literature, including longitudinal mentoring, situated participation in authentic clinical practice, and progressive assumption of responsibility under supervision [23, 32, 42, 43]. This definition provided a consistent basis for identifying apprenticeship models across specialties and institutional structures. Studies were eligible if they addressed apprenticeship features and PIF, mentoring, or the cost of caring. We included peer-reviewed articles and grey literature published in English between January 1, 2000 and December 30, 2025. Full inclusion and exclusion criteria are detailed in Table 1.

**Table 1:** Inclusion and exclusion criteria

PCC	Inclusion Criteria	Exclusion Criteria
Population	- Junior physicians, house officers, postgraduate year one physicians - Medical officers, senior medical officers, registrars, specialist registrars - Residents, senior residents, clinical fellows - Undergraduate and postgraduate medical students	- Allied health specialties, such as dietetics, nursing, psychology, chiropractic, midwifery, social work - Non-medical specialties, such as clinical and translational science, veterinary, dentistry
Concept	- Apprenticeship in medical education, its effect on PIF and similarities with mentoring - Effects of burnout, CF, VT, STS within the setting of medical education and apprenticeship	- Any paper that does not discuss apprenticeship
Context	- Medical education	

### The Modified Systematic Evidence-Based Approach (mSEBA)

Analysis proceeded through five stages, with an expert team of medical educators and clinicians (n=5) providing oversight throughout. This triangulated approach addressed the volume and heterogeneity of included studies across specialties, training levels, and apprenticeship models.

#### Stage 1: systematic search and selection

Two researchers (WTWL, VM) independently searched PubMed, Embase, ERIC, Scopus and Google Scholar using terms related to apprenticeship, medical education, PIF, mentoring, and cost of caring. A medical librarian reviewed the search strategy. The searches were conducted between 15 January 2026 to 24 January 2026. The full search strategy is provided in Appendix S2.

Abstracts were independently screened and duplicate records were removed by two reviewers (PA, VM) using EndNote 20. The abstracts were entirely co-screened by both reviewers. Disagreements were resolved through discussion, with a third reviewer (LKRK) adjudicating unresolved conflicts. Eligible articles underwent full-text review (WTWL, VM). Key findings from each included article were summarized in a structured data extraction table (see Appendix S3). 

##### AI-assisted searches

AI-assisted searches were used to identify eligible articles that might have been missed in manual database searches, thereby extending the scope of the searches given manpower constraints. To supplement manual searches, Elicit AI (Elicit, Inc., accessed June 2025) was used with identical search parameters based off the PCC criteria. One researcher (PA) reviewed the 92 articles identified by Elicit AI and selected 20 eligible articles to undergo full-text reviews.

#### Stage 2: split approach

Three independent teams analyzed included articles. Team 1 (PA, VM) conducted thematic analysis using Braun and Clarke’s approach [44, 45]. Team 2 (WTWL) performed directed content analysis using Hsieh and Shannon’s approach [46, 47], applying codes derived from apprenticeship literature [32, 42, 43]. Team 3 (LKRK) used ChatGPT 5 Pro (OpenAI, accessed June 2025) to compare and check codes identified by Teams 1 and 2. Consensus was achieved through negotiated validation, in which team members discussed and defended their interpretations [48, 49].

#### Stage 3: jigsaw perspective

Complementary themes from the three teams were merged using meta-ethnographic synthesis principles. Five key themes emerged from the analyses (Table 2).

**Table 2:** Key themes identified from analysis

Approach	Thematic Analysis	Direct Content Analysis
Key Themes and Categories	Socialization of apprentices Situated learning Critical reflection Impact on supervisor Limitations of apprenticeship	Socialization of apprentices Situated learning Critical reflection Impact on supervisor

#### Stage 4: verification

Merged themes were checked against article summaries for accuracy. Themes were examined iteratively to identify patterns relevant to PIF, mentoring relationships, and physician wellbeing.

#### Stage 5: AI-aided comparison

ChatGPT 5 Pro was used to compare themes from data-driven literature (empirical studies) and non-data-driven literature (opinion pieces and editorials) using directed content analysis categories [46]. This AI-supplemented analysis was conducted on 27 June 2025. Results were similar, suggesting minimal bias from non-data-driven sources.

##### AI transparency

ChatGPT 5 Pro was used to compare coding patterns and literature types. A sample of the chat conversation with ChatGPT 5 Pro is enclosed in Appendix S4. The prompts featured in Appendix S4 were replicated for each article uploaded in groups of ten. The use of AI tools served to streamline the comparison process by consolidating themes and organizing the data drawn from the manual analyses. All analytic decisions remained under human judgment. AI outputs were validated through five steps: comparison with manual coding, acceptance of concordant codes, senior review of discordant codes, verification of new AI codes against source articles, and retention of only the validated codes. All AI suggestions required manual verification and human oversight before inclusion.

## Results

**Fig. 3:**
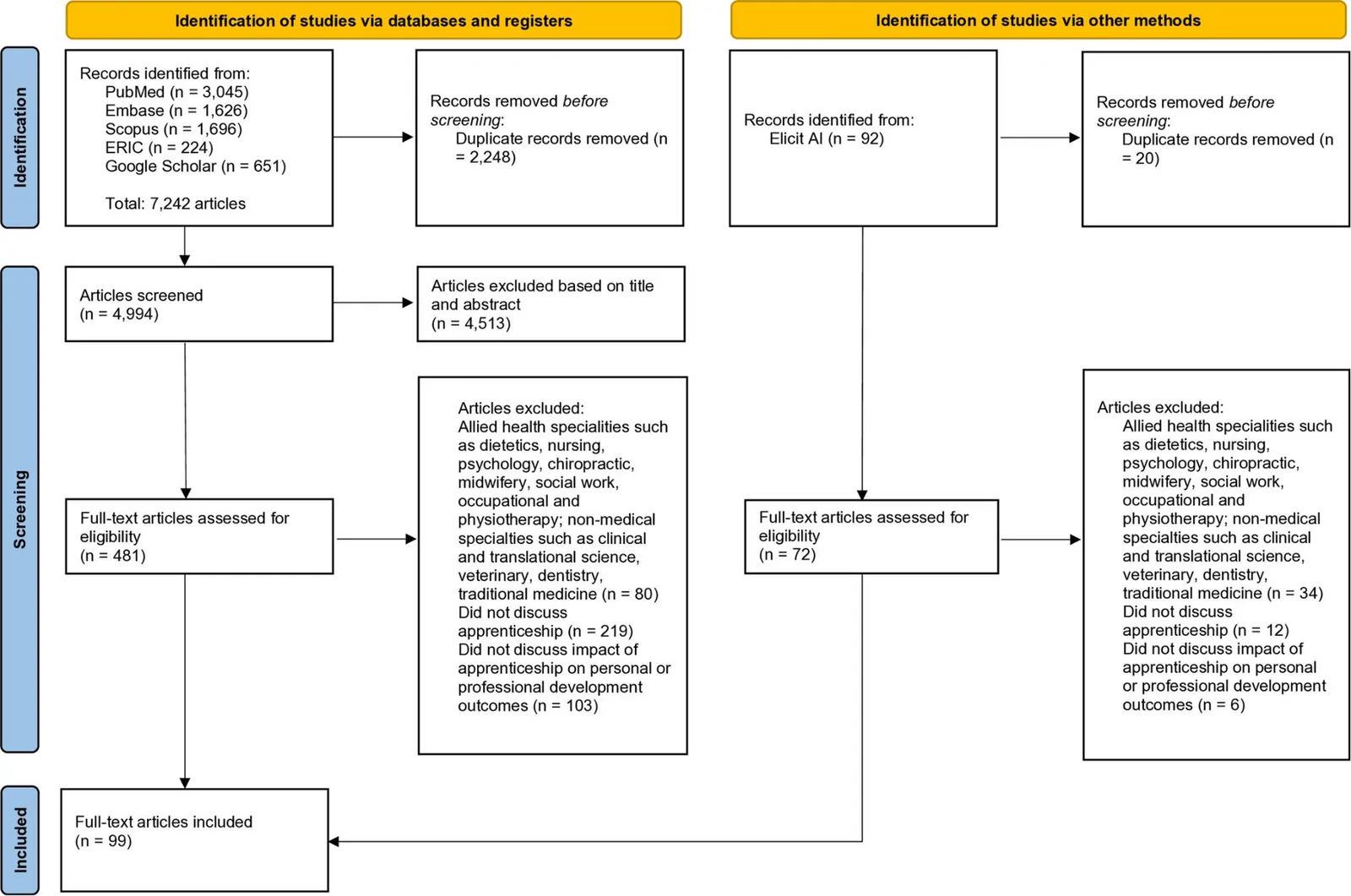
PRISMA flowchart. *Adapted from* http://www.prisma-statement.org/

The search strategy identified 7,242 articles from databases and 92 articles from Elicit AI, totaling 7,334 articles for screening. Of these, 481 database articles and 72 Elicit AI articles underwent full-text review. 99 articles met the inclusion criteria and were included in the final analysis (Fig. 3). Appendix S3 features a consolidated summary table of the included articles.

### Study characteristics

The 99 included studies represented diverse medical specialties, including palliative care, oncology, emergency medicine, and surgery. Training settings included hospital wards, outpatient clinics, operating theaters, and community-based practices. Program durations ranged from several months to multiple years. Included studies spanned all stages of medical training, from undergraduate medical students to registrars and fellows. The citation numbers for the 99 included articles are between numbers 50 to 148 in the reference list. The citation numbers for each article are also included in Appendix S3.

Analysis identified five key themes (Table 2), presented below.

### Theme 1: socialization of apprentices

Programs facilitated trainees’ transition from peripheral observation to central participation in clinical teams [50–60], with clear expectations regarding competencies and professional conduct. Early work focused on role modelling and supervised participation as the primary mechanisms driving this progression [58, 61–63]. Research in the 2010s broadened this understanding, examining how specific program structures, including learning communities, longitudinal clerkships, and assistantships, shaped professional values internalization and workplace identity development across diverse specialties and settings [50, 52, 64–66]. More recent work has shifted attention toward the relational conditions that enable or impede socialization, particularly the quality of supervisory relationships and the extent to which trainees experience meaningful participation rather than peripheral observation [55, 57, 67].

Community of practice (CoP) structures supported professional relationships [51, 53, 55, 57, 60, 61, 64, 65, 68–70] and fostered a sense of belonging [57, 62, 65, 71–73]. Participation in clinical teams enabled internalization of professional values and norms [57, 61, 63, 67, 69, 74–77]. Trainees reported clearer professional roles and values [50, 52, 53, 57, 59, 75, 76], growing identification with the profession [50, 53, 66] and workplace identity development as responsibility and autonomy increased [51, 53, 55, 66, 72, 78].

#### Burdens affecting socialization

Identity-related challenges included role ambiguity and trainees feeling they “fell through the cracks” [78] because of inadequate support and supervision, unclear expectations, lack of structure, and limited opportunities for meaningful participation [50, 72, 74, 76, 78, 79].

### Theme 2: situated learning

Early studies established that learning embedded in authentic clinical work, through participation in ward rounds, outpatient consultations, and procedural training, was central to both skill acquisition and professional development in apprenticeship settings [58, 61–63, 80, 81]. Research in the 2010s diversified this understanding considerably, examining how specific apprenticeship formats, including cognitive apprenticeship, coaching models, continuity clinics, and blended learning, shaped learning outcomes across a range of specialties and training levels [52, 53, 82–88]. More recent studies have examined the conditions under which situated learning is most effective, with particular attention to psychological safety, deliberate practice, and near-peer relationships as enabling factors [89–94].

Training occurred across hospital wards [52, 61, 71, 89], outpatient clinics [51, 53, 55, 63, 69, 82, 95], operating theaters [54, 78, 96] and continuity experiences [51, 57, 63, 66, 69, 82]. Longitudinal integrated clerkships provided sustained engagement in clinical environments [57, 66, 97], continuity clinics [51, 63], preceptorships [71, 80, 98] and job-shadowing programs [75, 76] offering extended supervised exposure to clinical practice. Some programs maintained apprenticeship principles through online or blended learning formats [83, 84, 99]. Across these varied settings, the defining feature distinguishing apprenticeship from general clinical exposure was the presence of structured mentored relationships within which trainees progressively assumed greater responsibility.

Role modeling involved observation and emulation of clinicians in an authentic clinical context [51, 58, 62, 63, 69, 72, 79, 89, 90, 100–103] while coaching provided guidance tailored to individual learner needs and clinical situations [83, 85, 86, 91, 104]. Programs incorporated supervised clinical practice with progressive responsibility [51, 52, 55, 56, 73, 105]. Situated learning was associated with improvements in clinical skills, diagnostic reasoning, and procedural competence [51–54, 57, 59, 62, 78, 83, 86, 89, 92], as well as the development of communication skills and patient-centered attitudes through sustained clinical immersion and simulation [52, 53, 62, 63, 66, 97, 106, 107].

#### Burdens in clinical settings

The apprenticeship settings documented in these studies were also contexts in which trainees encountered significant psychological burdens. Moral distress was documented specifically among trainees in surgical and pediatric programs, arising from value conflicts and the perceived inability to provide optimal care [108–110]. Compassion fatigue was reported particularly in palliative care, oncology, and emergency settings [79, 111, 112]. Vicarious trauma and secondary traumatic stress were documented among trainees with repeated exposure to distressing clinical events within training environments [79, 113, 114]. Burnout was documented at higher prevalence in high-intensity training environments, associated with both workload demands and the quality of mentoring relationships [87, 93, 114–116].

### Theme 3: critical reflection

Despite its recognized importance in apprenticeship, critical reflection has historically been more assumed than systematically supported, with early studies documenting that preceptor-facilitated discussion of difficult encounters occurred inconsistently and often informally [58, 62, 108, 117]. The 2010s saw growing efforts to formalize reflective practice [118], introducing structured debriefing, narrative reflection, and peer support groups, with evidence that these approaches improved emotional processing, ethical reasoning, and tolerance of uncertainty [52, 69, 72, 83, 86, 87, 119, 120]. More recent work has examined the relational conditions that make genuine reflection possible, identifying psychological safety, cognitive apprenticeship, and near-peer learning as enabling factors without which reflective structures remain superficial [59, 71, 90, 91, 93, 99, 109, 121, 122].

Feedback from trained mentors enhanced learner performance across a range of clinical and professional domains [59, 69, 71, 72, 83, 86, 91, 99, 104, 117]. Reflection provided structured opportunities for trainees to process experiences, examine emotional responses, and integrate learning [58, 69, 71, 101]. Guided reflection increased awareness of learning needs, strengths, limitations, and emotional responses [52, 59, 67, 121], while trainees reported improved ability to manage emotional reactions to challenging encounters, navigate uncertainty, and deliver difficult information [62, 79]. Programs that incorporated discussion of moral challenges supported the development of ethical reasoning [108–110, 119], and mentored reflection and peer support were associated with improved psychological outcomes [59, 90, 120, 122].

#### Effects on burdens

Mentor-facilitated discussion of ethical dilemmas and morally distressing encounters was associated with reduced moral distress and greater preparedness for ethical decision-making, including a reported reduction in perceived moral distress following structured ethics case discussion series [79, 109]. Mentor-led reflection and debriefing after emotionally intense clinical encounters were associated with reduced burnout indicators, with stronger supervisory alliances linked to lower distress and higher self-esteem among residents [79, 87, 93, 109, 114, 116]. Structured peer support and guided processing of empathically demanding encounters were associated with preserved empathy and reduced compassion fatigue in high-burden specialties [111]. Conversely, programs that lacked structured reflective components or provided inadequate debriefing after distressing encounters were associated with poorer psychological outcomes and greater vulnerability to vicarious trauma [79, 113].

### Theme 4: impact on supervisor

The supervisor has always been central to apprenticeship, yet the literature treating supervisors as subjects of study in their own right, rather than simply as delivery mechanisms for trainee learning, developed relatively late. Early studies focused primarily on how supervisor characteristics shaped trainee outcomes [117, 123–126]. It was not until the 2010s that researchers began examining the conditions that enable or constrain effective supervision, including mentoring environment quality, faculty development, and the structural demands placed on supervisors within increasingly pressured clinical systems [53, 71, 76, 101, 127–130]. More recent work has raised concern about the sustainability of the supervisory role itself, as inadequate institutional support for teaching threatens the quality and continuity of apprenticeship models [131, 132].

Effective apprenticeship was consistently found to depend on trained faculty and mentors, with mentoring requiring skills in feedback delivery, adaptive teaching, and responsiveness to individual learner needs [53, 59, 71, 75, 83, 85, 89, 91, 92, 104, 117, 123, 127, 133]. Mentor-mentee relationship quality was consistently identified as a key determinant of apprenticeship effectiveness, influencing trainees’ professional identity development, confidence, and capacity to manage clinical challenges [53–55, 57, 59, 67, 69, 71, 72, 76, 78, 85, 127]. Mentors described both the intrinsic rewards of teaching and the emotional demands of supporting trainees through difficult clinical experiences [58, 104, 131].

Alongside these relational demands, supervisors faced significant structural challenges in fulfilling their teaching roles, including time constraints, competing clinical demands, and limited protected teaching time [71, 76, 78, 79, 101, 134, 135]. Faculty development in mentoring skills was inconsistent across programs, with variation in supervisor training contributing to uneven apprenticeship quality within and across institutions [76, 101, 104]. Where supervision was perceived as inadequate, trainees reported reduced engagement, diminished sense of belonging, and greater vulnerability to identity-related challenges [53, 72, 76, 78] .

#### Burdens affecting supervisors

Mentors described vicarious distress arising from supporting trainees through morally and emotionally challenging encounters, as well as the cumulative burden of sustained emotional investment in multiple mentoring relationships [58, 104]. Time pressure and role conflict between clinical and teaching responsibilities contributed to supervisor stress and, in some cases, reduced capacity for meaningful mentoring engagement [71, 76, 126, 131].

### Theme 5: limitations of apprenticeship

The limitations of apprenticeship are not a recent discovery. Concerns about variability in clerkship quality, inconsistent assessment, and the challenge of standardizing learning across diverse clinical environments were present in the earliest literature in this review [61, 80, 123–125, 136]. What has changed over time is not the nature of these concerns but their urgency, as training reforms, workforce pressures, and shifts toward competency-based education have intensified longstanding structural tensions [52, 53, 73, 74, 82, 95, 127, 128, 137–140]. More recent work has added new dimensions to this picture, examining cultural diversity, psychological safety, and workforce sustainability as challenges that existing apprenticeship models are not consistently equipped to address [89, 90, 131, 141–147].

Structural limitations of apprenticeship have been consistently identified across the literature, including concerns about standardization, sustainability, and fitness for purpose in contemporary medical education [126, 128–130]. Assessment of apprenticeship quality and outcomes remained heterogeneous, limiting cross-study comparisons [52, 53, 56, 73, 79, 80, 91, 117, 123–125, 137]. Variability in program structure, duration, and components also challenged standardization [60, 71, 84, 141]. Time and workload constraints on both supervisors and trainees limited the depth and consistency of mentoring relationships across programs [71, 76, 78, 79, 101, 126].

Evidence gaps were also identified. Few studies examined relational aspects of physician development, including peer relationships and family dynamics [55, 57, 117, 148]. Long-term outcomes of apprenticeship programs remain understudied, with most evidence limited to training period effects [52, 55, 56, 84]. Most included studies were conducted in Western, English-language contexts, limiting generalizability to diverse cultural settings where concepts of professional identity, mentorship, and the supervisory relationship may differ substantially [127, 142]. Notably, general practice (GP), a specialty with a longstanding apprenticeship tradition and documented high rates of burnout and compassion fatigue, was underrepresented in the included literature, with only two studies examining GP-based apprenticeship in depth [55, 57]. Direct empirical evidence linking specific apprenticeship mechanisms to the cost of caring elements described in this review was limited; most included studies examined professional development outcomes rather than psychological burden outcomes specifically, and the connections proposed in this review are largely theoretically informed.

## Discussion

Apprenticeship adds a deliberate developmental architecture to general clinical exposure, one that explicitly shapes professional identity in ways that clinical work alone cannot reliably achieve. Through longitudinal mentored relationships, progressive entrustment, structured reflective practice, and guided socialization into a community of professional practice, apprenticeship creates the conditions for professional identity to be actively supported rather than left to chance.

The five themes identified, socialization, situated learning, critical reflection, supervisor impact, and the limitations of apprenticeship, describe not five separate findings but an interconnected system. Socialization provides the entry point, through which trainees begin to form professional identities within CoPs [50, 60, 64, 65, 68]. Situated learning provides the context, embedding skill acquisition and values development in authentic clinical work [51–54, 57, 59, 62, 78, 89]. Critical reflection provides the processing mechanism, through which difficult experiences are examined and integrated rather than simply accumulated [58, 69, 71, 101, 108–110, 119]. The supervisor provides the relational anchor whose availability, skill, and own wellbeing determines whether this system functions or fails [53, 59, 71, 117, 123, 127]. Where structural failures, assessment gaps, or cultural constraints undermine these elements, the system breaks down and with it, the protection apprenticeship may otherwise afford [61, 80, 123, 128–130].

Drawing on 99 included studies, specific associations were identified between apprenticeship features and cost of caring outcomes. Mentor-facilitated discussion of moral challenges and structured ethics case series were associated with reduced moral distress and greater preparedness for ethical decision-making [79, 108, 109]. Structured peer support and mentor-led reflection were associated with reduced emotional burden in high-demand specialties, including reported reductions in compassion fatigue and empathic erosion [109, 111, 114]. Stronger supervisory alliances and mentoring relationships were associated with lower burnout and distress among trainees [87, 93]. These associations, while modest in scope and largely drawn from studies not designed to test cost of caring outcomes directly, provide the empirical foundation for the theoretical interpretation that follows.

### Theoretical framework application

The Ring Theory of Personhood (RToP) offers a lens through which to understand where apprenticeship mechanisms may be operating within the structure of personhood. Analysis of the included studies suggests that most research has examined what the RToP would characterize as the Societal Ring, focusing on professional development outcomes including professional socialization, competency acquisition, and role transition. Fewer studies examined the Individual Ring, addressing individual psychological states, and very few examined outcomes corresponding to the Innate Ring, including core beliefs and sense of calling. This distribution suggests the field has examined the outer dimensions of personhood most extensively, leaving the deeper levels of personhood and identity protection largely uninvestigated. Through the RToP lens, disruptions that penetrate to the Innate Ring, eroding a physician’s sense of calling and core professional values, may represent the most damaging manifestations of the cost of caring, yet these are precisely the outcomes least represented in the current evidence base.

Within this framework, the apprenticeship features documented across the five themes may be understood as operating across multiple rings simultaneously. CoP structures and progressive socialisation may reinforce professional identity at the Societal Ring while providing the collegial relationships that sustain the Relational Ring [60, 64, 65, 68]. Guided reflection and mentored meaning-making may protect the Individual Ring by preventing the accumulation of unprocessed emotional distress [58, 67, 69, 101, 127]. Role modeling by experienced physicians who demonstrate how to integrate emotionally demanding work with sustained professional values may help preserve the Innate Ring, affirming trainees’ sense of calling even under conditions of significant burden [58, 67, 72, 89, 100, 101]. Continuity of mentoring relationship emerged as a consistent differentiating feature across the included studies, with programs providing sustained longitudinal mentoring associated with stronger identity development outcomes than those relying on supervision without continuity [50, 55, 57, 66, 69, 97]. Where apprenticeship mechanisms are absent or inadequate, the evidence suggests that disruptions may cascade across rings, with burnout, compassion fatigue, and moral distress reflecting failures at multiple levels of personhood simultaneously [87, 93, 111, 113, 116].

The Internal Compass framework offers a complementary lens through which to understand the developmental mechanisms apprenticeship may support. The five Internal Compass components, self-awareness, clinical judgment, ethical reasoning, professional confidence, and autonomous decision-making, each correspond to specific apprenticeship features documented in the included studies. Guided reflection and individualized debriefing were associated with enhanced self-awareness of emotional responses and personal limitations [52, 59, 67, 121]. Situated learning and progressive clinical responsibility were associated with the development of clinical judgment and professional confidence [51–54, 57, 59, 62, 78, 89]. Mentored discussion of moral challenges and structured ethics case discussions were associated with improved ethical reasoning and reduced moral distress [79, 108–110]. Scaffolded independence under supervision was associated with the development of autonomous decision-making capacity [51, 52, 55, 56, 73, 105]. We propose that apprenticeship, through these mechanisms, supports the progressive maturation of the Internal Compass, and that physicians with a well-developed Internal Compass may be better equipped to navigate the emotional and moral demands of clinical work without lasting disruption to their professional identity. Where the Internal Compass remains underdeveloped, through inadequate supervision, absent reflective practice, or poorly supported socialisation, physicians may be more vulnerable to the cumulative burden of the cost of caring [2, 3]. This proposition, while theoretically grounded in the included evidence, requires direct empirical validation.

### Evidentiary considerations

When interpreting these propositions, it is important to acknowledge that direct empirical evidence linking specific apprenticeship mechanisms to cost of caring outcomes was limited in the included literature. Most included studies were designed to examine professional development outcomes rather than psychological burden outcomes specifically, and the associations between apprenticeship features and cost of caring protection reported in this review largely reflect theory-informed synthesis rather than direct empirical demonstration. The limited direct evidence is itself informative, mapping the current state of the field and identifying a specific gap for future research. The synthesis presented here offers a basis for hypothesis generation and intervention design, but the proposed causal mechanisms require testing through longitudinal studies with appropriately designed outcome measures.

### Implications for practice

For institutions seeking to establish or strengthen apprenticeship models, the evidence suggests that the quality of mentoring relationships, the availability of structured reflective practice, and the protection of supervisor time are the highest-leverage elements. Investment in faculty development, equipping supervisors with skills in feedback delivery, adaptive teaching, and responsiveness to individual trainee needs, is likely to be more effective than expanding clinical exposure alone [53, 59, 71, 117, 123, 127]. Structured debriefing after emotionally demanding encounters, ethics case discussion series, and peer support mechanisms should be considered core components of apprenticeship programs rather than supplementary additions, given their documented associations with reduced moral distress, preserved empathy, and improved psychological outcomes [79, 90, 108–111]. These elements, taken together, constitute the developmental architecture that distinguishes effective apprenticeship from general clinical placement, and their intentional design and protection are what the evidence most strongly supports.

For settings where full apprenticeship models are not currently feasible, the themes suggest that targeted implementation of key apprenticeship features may still provide meaningful protection against cost of caring elements. Structured reflective practice and continuity of at least one mentored relationship may be achievable even within resource-constrained environments, and represent minimum viable components of the apprenticeship architecture described in this review. Settings already operating apprenticeship models should attend particularly to the sustainability of the supervisory role. The evidence suggests that supervisor burden and role conflict represent vulnerabilities that can undermine the entire system, and that institutional protection of teaching time is not merely an organisational preference but a prerequisite for effective apprenticeship delivery [71, 76, 126].

### Limitations & future directions

This review has several limitations. The heterogeneity of apprenticeship models and outcome measures across the 99 included studies limits direct comparisons and firm conclusions about which components are most protective. Most included studies employed observational designs, precluding causal inference [149]. Studies predominantly represented Western, English-language contexts, limiting generalizability to diverse cultural settings where concepts of personhood, professional identity, and mentorship may differ substantially [150]. Long-term outcomes remained understudied, with most evidence limited to effects observed during training; whether protective effects persist into independent practice is unknown. Included studies spanned all stages of medical training, from undergraduate students through to residents, registrars, and fellows, and findings should be interpreted with this heterogeneity in mind [151]. General practice, a specialty with a longstanding apprenticeship tradition and documented high rates of burnout and compassion fatigue, was underrepresented in the included literature, with only two studies examining GP-based apprenticeship in depth [55, 57]. The use of AI tools in the search and analytical stages of this review, while consistent with emerging practice and transparently reported, introduces limitations around reproducibility that more traditional search methodologies do not [152]. Finally, as noted earlier, direct empirical evidence linking specific apprenticeship mechanisms to cost of caring outcomes was limited; the theory-informed synthesis presented here requires empirical validation.

Future research should prioritise longitudinal study designs that track both professional development and psychological burden outcomes across training and into independent practice, enabling more direct testing of the causal mechanisms proposed in this review. Studies specifically examining the relationship between apprenticeship features and Internal Compass development, and between Internal Compass maturity and cost of caring outcomes, would provide the empirical foundation that the current literature lacks. Cross-cultural research is essential to determine whether the RToP and Internal Compass frameworks apply across diverse contexts or require cultural adaptation [142]. General practice represents a particularly important and underexplored context for this research agenda, given its strong apprenticeship tradition, its global reach, and the documented vulnerability of its workforce to burnout and compassion fatigue [55, 57]. Research examining the differential effects of apprenticeship across training stages, from undergraduate students through to residents, registrars, and fellows, would address the heterogeneity limitation identified in this review and clarify whether apprenticeship principles operate consistently across the continuum of medical education or whether stage-specific approaches are warranted.

AI tools can be used in future studies as an aid to identify potential research missed due to human oversight. Our review illustrates the usefulness of AI-supplemented approaches in systematic scoping reviews that feature large volumes of data, especially given resource and manpower constraints. AI-supplemented approaches should acknowledge the tendency to bias highly cited studies over emerging research [153] and should thus be integrated into a hybrid methodology, such as mSEBA, that prioritizes human decision-making and verification.

More broadly, the RToP framework suggests that interventions targeting isolated dimensions of physician wellbeing, such as burnout or empathy preservation in isolation, may be insufficient. Future intervention studies should consider designs that support physicians across multiple dimensions of personhood simultaneously, examining whether whole-person approaches produce more durable protection against the cost of caring than symptom-specific interventions.

## Conclusion

The cost of caring poses a significant and sustained challenge to physicians in emotionally demanding specialties, with consequences for both individual wellbeing and the sustainability of the workforce. This systematic scoping review mapped the evidence on apprenticeship models and their relationship to these burdens, synthesizing findings from 99 included studies through the theoretical lenses of the RToP and the Internal Compass. The five themes identified, socialization, situated learning, critical reflection, supervisor impact, and the limitations of apprenticeship, describe an interconnected developmental system that, when functioning well, may address professional identity disruption at multiple levels simultaneously. Direct empirical evidence linking specific apprenticeship mechanisms to cost of caring outcomes remains limited, and the connections proposed in this review are largely theory-informed. Concurrent manual and AI-supported analyses enhanced the interpretation of various apprenticeship models through the lens of the RtoP and Internal Compass frameworks, addressing the blind spots presented from the complementary analyses conducted. The synthesis presented here offers a theoretical foundation for hypothesis generation, intervention design, and future empirical research. Institutions investing in apprenticeship should prioritize the quality of mentoring relationships, structured reflective practice, and the sustainability of the supervisory role as the highest-leverage elements of an effective developmental architecture. 

## Supplementary Information

Supplementary Material 1.

Supplementary Material 2.

Supplementary Material 3.

Supplementary Material 4.

## Data Availability

All data generated or analyzed during this study are included in this published article and its supplementary information files.

## Abbreviations

RToP: Ring Theory of Personhood
PIF: Professional identity formation
mSEBA: Modified Systematic Evidence-Based Approach
CoP: Community of Practice
