# Supplementary material for: The role of physician apprenticeship models in the cost of caring: an AI-assisted systematic scoping review
Source: BMC Med Educ. 2026 Jul 27;26:1706. doi: 10.1186/s12909-026-09932-y (PMC13692915; doi:10.1186/s12909-026-09932-y)
Supplement: Supplementary file 2 [file 12909_2026_9932_MOESM2_ESM.pdf]

44. Affirming Professiona...
PDF

Please analyse the following article and provide relevant verbatim statements that support these points. If there are no relevant mention of the points, please say so: 1) Studies described trainees reporting clearer sense of professional role and values. 2) Trainees described growing identification with the profession. 3) Role ambiguity was reported in studies of junior physicians experiencing sustained distress without adequate support. 4) findings on Community of practice
 Professional values internalization
 Role transition from observation to participation
 Professional identity development
 Identity-related challenges 5)

- **Article analysed**
  - From McGill University: *Affirming Professional Identities Through an Apprenticeship: Insights From a Four-Year Longitudinal Case Study* (Academic Medicine, 2014). 44. Affirming Professional Iden...
  - Note on scope (re: your Point 3): this is an **undergraduate medical student** apprenticeship study (not a study of junior physicians/residents). 44. Affirming Professional Iden...

- **1) Trainees reported clearer sense of professional role and values**
  - **Professional role clarity (student)**
    - "You're sort of representing a school, a hospital, a profession as a whole." (Boudreau, Macdonald, & Steinert, 2014, p. 1041) 44. Affirming Professional Iden...
  - **Clearer sense of "being physicians" (teacher witnessing student role-formation)**
    - "more of a sense of being physicians...." (Boudreau et al., 2014, p. 1041) 44. Affirming Professional Iden...
  - **Values clarity / values language (teacher describing student capability)**

        - “There can be a certain set of values.” (Boudreau et al., 2014, p. 1041) 44. Affirming Professional Iden...
        - “you bump up against something that’s against those values and you’re able to say, ‘This was kind of against those values.’” (Boudreau et al., 2014, p. 1041) 44. Affirming Professional Iden...
    - **Explicit “who I want to be” professional-self evaluative framing (student)**
        - “What is being a clinical clerk doing to us; am I turning into the doctor I want to be?” (Boudreau et al., 2014, p. 1041) 44. Affirming Professional Iden...

- **2) Trainees described growing identification with the profession**
    - **Growing identification / inseparability from professional identity (student)**
        - “I find more and more I’m sort of intertwined with my profession and you can’t escape it.” (Boudreau et al., 2014, p. 1041) 44. Affirming Professional Iden...
    - **Identity “enmeshment” (authors’ analytic statement, grounded in participant narratives)**
        - “Professional and personal identities became enmeshed, to the point that they were difficult to disentangle.” (Boudreau et al., 2014, p. 1042) 44. Affirming Professional Iden...
    - **Belonging/affiliation framing (authors’ results summary)**
        - “Students became increasingly aware of having entered the kinship of physicians.” (Boudreau et al., 2014, p. 1038) 44. Affirming Professional Iden...

- **3) Role ambiguity + sustained distress without adequate support in junior physicians**
    - **Junior physicians with sustained distress + inadequate support**
        - **No relevant mention found** in this article that specifically reports **junior physicians** experiencing **sustained distress without adequate support**. 44. Affirming Professional Iden...
    - **Role ambiguity-like transitional uncertainty (student; *medical student* not junior physician)**
        - “I feel like I’m somewhere in between.” (Boudreau et al., 2014, p. 1042) 44. Affirming Professional Iden...
    - **Distress/need for support (but not “without support”; rather, the apprenticeship is framed as supportive)**
        - “There are students who desperately need help and won’t speak to me.” (Boudreau et al., 2014, p. 1041) 44. Affirming Professional Iden...

- "It's more some kind of emotional support…" (Boudreau et al., 2014, p. 1041) 44. Affirming Professional Iden…

- **4) Verbatim statements relevant to: community of practice; values internalization; role transition; professional identity development; identity-related challenges**
  - **Community of practice**
    - (Conceptual framing) "Its sociocultural characteristics are encapsulated in the notion of 'communities of practice.'" (Boudreau et al., 2014, p. 1038) 44. Affirming Professional Iden…
    - (Findings/interpretation consistent with CoP-like belonging) "buttress and nurture the progressive entry of novices into the kinship of medical professionals." (Boudreau et al., 2014, p. 1044) 44. Affirming Professional Iden…
    - (Relational-social metaphor aligning with a community/kinship construct) "The metaphor of family or kinship may be appropriate." (Boudreau et al., 2014, p. 1043) 44. Affirming Professional Iden…
  - **Professional values internalization**
    - "There can be a certain set of values." (Boudreau et al., 2014, p. 1041) 44. Affirming Professional Iden…
    - "you bump up against something that's against those values and you're able to say, 'This was kind of against those values.'" (Boudreau et al., 2014, p. 1041) 44. Affirming Professional Iden…
    - "Teachers experienced a renewal and validation of their commitment to the ideals of medicine." (Boudreau et al., 2014, p. 1038) 44. Affirming Professional Iden…
  - **Role transition from observation to participation**
    - (Early orientation to observation) "I think observing a senior physician will help to teach me my expectations." (Boudreau et al., 2014, p. 1040) 44. Affirming Professional Iden…
    - (Movement toward participation) "more of an active participant than a passive recipient." (Boudreau et al., 2014, p. 1041) 44. Affirming Professional Iden…
  - **Professional identity development**
    - (Explicit identity-formation aim) "particularly its contribution to professional identity formation." (Boudreau et al., 2014, p. 1038) 44. Affirming Professional Iden…
    - (Identity as emergent) "an evolving, albeit inchoate, identity:" (Boudreau et al., 2014, p. 1042) 44. Affirming Professional Iden…
    - (Transitional identity location) "I feel like I'm somewhere in between." (Boudreau et al., 2014, p. 1042) 44. Affirming Professional Iden…

- (Authors' conclusion about identity formation) "can contribute to the formation and reaffirmation of professional identity." (Boudreau et al., 2014, p. 1044) 44. Affirming Professional Iden...
- **Identity-related challenges**
  - (Mismatch expectations / early dissatisfaction) "students' dissatisfaction surfaced." (Boudreau et al., 2014, p. 1040) 44. Affirming Professional Iden...
  - (Authenticity concerns) "They became frustrated with the paucity of clinical experiences." (Boudreau et al., 2014, p. 1040) 44. Affirming Professional Iden...
  - (Perceived inauthenticity of early role) "Some qualified their relationship with the first-year patient as 'false,'" (Boudreau et al., 2014, p. 1040) 44. Affirming Professional Iden...
  - (Emotional coping as part of identity work) "You try to retain compassion but you do kind of start developing more of a—not a wall, but like a kind of coping mechanism." (Boudreau et al., 2014, p. 1041) 44. Affirming Professional Iden...

- **5)**
  - **No Point 5 content was provided in your prompt**, so I cannot extract supporting verbatim statements for it. 44. Affirming Professional Iden...

- **Reference (APA 7th)**
  - Boudreau, J. D., Macdonald, M. E., & Steinert, Y. (2014). Affirming professional identities through an apprenticeship: Insights from a four-year longitudinal case study. *Academic Medicine, 89*(7), 1038–1045. 44. Affirming Professional Iden...

Sources

29. Exploring the hidden ...
PDF

Please analyse the following article and provide relevant verbatim statements that support these points. If there are no relevant mention of the points, please say so: 1) Studies described programs as facilitating internalization of professional values and norms through participation in clinical teams. 2) Role ambiguity was reported in studies of junior physicians experiencing sustained distress without

adequate support. 3) Studies identified several limitations of apprenticeship approaches beyond supervisor constraints. 4) Variability in program structure, duration, and components challenged standardization efforts. 5) Community of practice
 Professional values internalization
 Role transition from observation to participation
 Professional identity development
 Identity-related challenges 6) Training across diverse settings (wards, clinics, theaters)
 Longitudinal structures (LICs, continuity clinics)
 Role modeling
 Coaching
 Supervised practice
 Clinical competency development

burnout) 7) Heterogeneous assessment approaches
 Program structure and duration variability
 Time and workload constraints
 Limited research on relational aspects
 Gaps in long-term outcomes
 Western context bias
 Limited focus on cost of caring mechanisms

Show less

Thought for 2m 54s

Article analysed

- Kittmer, T., Hoogenes, J., Pemberton, J., & Cameron, B. H. (2013). *Exploring the hidden curriculum: A qualitative analysis of clerks' reflections on professionalism in surgical clerkship*. **The American Journal of Surgery, 205**, 426–433. 29. Exploring the hidden curric...

---

1) Programs facilitating internalization of professional values and norms through participation in clinical teams

- **Progressive participation in clinical teams**

  - "As medical students enter clerkship, they begin to participate progressively in the care of patients with mentors and other health professionals" (Kittmer et al., 2013, p. 426).
- **Learning professionalism through everyday team practices (hidden curriculum)**
  - Students "compare and contrast the behaviors of their teachers and peers with the professional ideals they have been taught" (Kittmer et al., 2013, p. 426).
- **Structured program reinforcing values**
  - "Group members reinforced one another's values and empowered each other in a reflective manner" (Kittmer et al., 2013, p. 427).
- **Values becoming enacted in practice**
  - "Those values they had merely talked about theoretically came alive in their everyday experiences" (Kittmer et al., 2013, p. 427).

---

2) Role ambiguity in junior physicians experiencing sustained distress without adequate support

- **Junior physicians (residents) with sustained distress**
  - **No relevant mention found**. The study population comprises **medical clerks**, not junior physicians or residents.
- **Role ambiguity and distress in clerks (not junior physicians)**
  - "Others simply were not clear on what their role was to be in certain situations" (Kittmer et al., 2013, p. 428).
  - "Clerk asked to round alone on patients on a weekend and feeling unsupported" (Kittmer et al., 2013, p. 430).

---

3) Limitations of apprenticeship approaches beyond supervisor constraints

- **Apprenticeship framing**
  - The article does **not explicitly conceptualize the clerkship as an 'apprenticeship'** model.
- **Limitations of the educational approach and study**
  - "More sensitive topics may have been underrepresented because the written CIRs had to be discussed in small groups" (Kittmer et al., 2013, p. 431).
  - "Because our sampling was limited to a single class year at a single institution, it is difficult to generalize" (Kittmer et al., 2013, p. 431).
  - "Our analysis did not involve coding the small group discussions" (Kittmer et al., 2013, p. 431).

---

4) Variability in program structure, duration, and components challenging standardization

- **Program variability**
  - **No relevant mention found**. The clerkship is described as a **mandatory, standardized 6-week rotation** with fixed components.

---

5) Findings related to key theoretical constructs

a) Community of practice

- **Implicit (not explicitly named)**
  - Clerks reflect on "their interactions with members of the health care team" and surgical culture (Kittmer et al., 2013, p. 428).

b) Professional values internalization

- "Clerks repeatedly reinforced the value of putting patients first" (Kittmer et al., 2013, p. 428).

c) Role transition from observation to participation

- "They begin to participate progressively in the care of patients" (Kittmer et al., 2013, p. 426).
- "Some clerks were given too much responsibility… others simply were not clear on what their role was" (Kittmer et al., 2013, p. 428).

d) Professional identity development

- CIRs are based on events that "influenced his/her professional development" (Kittmer et al., 2013, p. 427).
- Clerks reflected on "their evolving professional self" (Kittmer et al., 2013, p. 428).

e) Identity-related challenges

- "Fear of losing opportunities… or getting a negative evaluation" constrained action (Kittmer et al., 2013, p. 431).
- "Verbal abuse also interferes with our ability to learn" (Kittmer et al., 2013, p. 428).

---

6) Educational features and burdens in clinical settings

a) Training across diverse settings

- Operating room: "in the operating room while the patient was still fully conscious" (Kittmer et al., 2013, p. 428).

- Clinic: “When we saw him in clinic, I was shocked” (Kittmer et al., 2013, p. 428).
- Emergency room: “interrupting the ER physician who was presenting the case” (Kittmer et al., 2013, p. 429).

b) Longitudinal structures (LICs, continuity clinics)

- **No relevant mention found**.

c) Role modeling

- Clerks “observed another physician dealing with a difficult issue in an exemplary way” (Kittmer et al., 2013, p. 427).

d) Coaching

- “Immediate, tangible feedback from peers and facilitators” supported learning (Kittmer et al., 2013, p. 431).

e) Supervised practice

- “Obtaining surgical consents… or rounding on ward patients unsupervised” (Kittmer et al., 2013, p. 428).

f) Clinical competency development

- Professionalism defined as “habitual and judicious use of… communication… values, and reflection in daily practice” (Kittmer et al., 2013, p. 426).

g) Burdens (moral distress, fatigue, burnout)

- Moral distress: clerks “suffered moral distress and were at a loss as to how to respond” (Kittmer et al., 2013, p. 430).
- Fatigue: “fatigue and a lack of sleep are correlated with poorer cognitive function” (Kittmer et al., 2013, p. 429).
- Burnout (suggested): “It is possible that senior clerk burnout may have played a role” (Kittmer et al., 2013, p. 431).

---

7) Broader methodological and contextual gaps

- **Heterogeneous assessment approaches**
  - “Papers are not marked” to promote safety (Kittmer et al., 2013, p. 427).
- **Program structure/duration variability**
  - **No relevant mention found**.
- **Time and workload constraints**
  - “Optimizing learning given time constraints” identified as a theme (Kittmer et al., 2013, p. 430).

- Extreme workload: "working 301 hours in surgery is acceptable" (Kittmer et al., 2013, p. 429).
- **Limited research on relational aspects**
  - "We are not aware of any literature that has examined the differences between junior and senior clerks' experiences" (Kittmer et al., 2013, p. 431).
- **Gaps in long-term outcomes**
  - **No relevant mention found**.
- **Western context bias**
  - **Not explicitly discussed** (single Canadian institution).
- **Limited focus on cost of caring mechanisms**
  - Moral distress discussed, but **no explicit analysis of long-term 'cost of caring' mechanisms** beyond immediate distress.

APA reference

- Kittmer, T., Hoogenes, J., Pemberton, J., & Cameron, B. H. (2013). Exploring the hidden curriculum: A qualitative analysis of clerks' reflections on professionalism in surgical clerkship. *The American Journal of Surgery, 205*(4), 426–433.

Sources

45. Applying the apprenti...
PDF

Please analyse the following article and provide relevant verbatim statements that support these points. If there are no relevant mention of the points, please say so: 1) Studies described trainees reporting clearer sense of professional role and values. 2) Studies identified several limitations of apprenticeship approaches beyond supervisor constraints. 3) Variability in program structure, duration, and components challenged standardization efforts. 4) Community of practice
 Professional values internalization
 Role transition from observation to participation
 Professional identity development
 Identity-related challenges 5) Heterogeneous assessment approaches
 Program structure and duration variability
 Time and workload constraints

- ▯ Limited research on relational aspects
- ▯ Gaps in long-term outcomes
- ▯ Western context bias
- ▯ Limited focus on cost of caring mechanisms

Show less

Thought for 3m 16s

Article analysed

- Nirodi, P., El-Sayeh, H., & Henfrey, H. (2018). *Applying the apprenticeship model to psychiatry: An evaluation*. **Progress in Neurology and Psychiatry, 22**(1), 25–29.
  45. Applying the apprenticeship...

---

1) Trainees reporting clearer sense of professional role and values

- **Clearer understanding of role / purpose**
  - "high scores in terms of students understanding the purpose of the scheme" (Nirodi et al., 2018, p. 26). 45. Applying the apprenticeship...
  - "Clear expectation of the week given" (student feedback, Table 3) (Nirodi et al., 2018, p. 28). 45. Applying the apprenticeship...
- **Professional values (patient-centredness, professionalism)**
  - "Emphasis on 'patient-centred care' and the experience of illness in addition to the disease process" (Nirodi et al., 2018, p. 26). 45. Applying the apprenticeship...
  - "allows students to assimilate the social norms and professional culture within which they reside" (Nirodi et al., 2018, p. 25). 45. Applying the apprenticeship...

---

2) Limitations of apprenticeship approaches beyond supervisor constraints

- **Time and resource demands**
  - "This requires investment of time and resources" (Nirodi et al., 2018, p. 25). 45. Applying the apprenticeship...
  - "mentors to be allowed protected time to dedicate to their apprentices" (Nirodi et al., 2018, p. 25). 45. Applying the apprenticeship...
- **Risks to patient care**
  - "the student requires close supervision… to ensure patient care is not compromised" (Nirodi et al., 2018, p. 28). 45. Applying the apprenticeship...
- **Methodological / study limitations**

- "small sample size" (Nirodi et al., 2018, p. 28). 45. Applying the apprenticeship...

---

3) Variability in program structure, duration, and components challenging standardization

- **Changes in duration**
  - "the scheme has now been extended to two weeks" (Nirodi et al., 2018, p. 29). 45. Applying the apprenticeship...
- **Changes in components / setting**
  - "the scheme has now been altered to be only within an inpatient or hospital-based team" (Nirodi et al., 2018, p. 29). 45. Applying the apprenticeship...
- **Explicit challenge to standardization**
  - **No explicit mention** that variability challenged standardization efforts; variability is described descriptively rather than as a policy or systems problem.

---

4) Findings mapped to key constructs

a) Community of practice

- "Each student is placed within a multidisciplinary team and encouraged to become involved in patient care" (Nirodi et al., 2018, p. 26). 45. Applying the apprenticeship...
- "Felt part of the team – rather than a spare part" (student feedback, Table 1) (Nirodi et al., 2018, p. 27). 45. Applying the apprenticeship...

b) Professional values internalization

- "allows students to assimilate the social norms and professional culture" (Nirodi et al., 2018, p. 25). 45. Applying the apprenticeship...
- "development of other skills such as communication and professionalism" (Nirodi et al., 2018, p. 25). 45. Applying the apprenticeship...

c) Role transition: observation → participation

- "The transition from passive observer to active member of the team" (Nirodi et al., 2018, p. 25). 45. Applying the apprenticeship...
- "after an initial period of shadowing the student will be expected to act as a team member" (Nirodi et al., 2018, p. 26). 45. Applying the apprenticeship...

d) Professional identity development

- "Great opportunity to practice skills to be skilled up more to become F1" (student feedback, Table 1) (Nirodi et al., 2018, p. 27). 45. Applying the apprenticeship...

- “Developed a further interest into the speciality – idea as a future career” (student feedback, Table 1) (Nirodi et al., 2018, p. 27). 45. Applying the apprenticeship...

e) Identity-related challenges

- “Expectation of me could be clearer” (student feedback, Table 2) (Nirodi et al., 2018, p. 27). 45. Applying the apprenticeship...
- “one week was not sufficient for them to experience being part of the team” (Nirodi et al., 2018, p. 28). 45. Applying the apprenticeship...

---

5) Broader methodological and contextual gaps

- **Heterogeneous assessment approaches**
  - Assessment described as “structured feedback in the form of written questionnaires” (Nirodi et al., 2018, p. 26); **no discussion of heterogeneity across programs**. 45. Applying the apprenticeship...
- **Program structure and duration variability**
  - “extended to two weeks” and “altered to be only within an inpatient... team” (Nirodi et al., 2018, p. 29). 45. Applying the apprenticeship...
- **Time and workload constraints**
  - “Arguments against the apprenticeship model based on lack of time and resources” (Nirodi et al., 2018, p. 25). 45. Applying the apprenticeship...
- **Limited research on relational aspects**
  - **No explicit statement** identifying relational aspects as under-researched; relationships are discussed but not framed as a research gap.
- **Gaps in long-term outcomes**
  - “to gain insight into the effects on future career choices” (Nirodi et al., 2018, p. 29), indicating outcomes not yet known. 45. Applying the apprenticeship...
- **Western context bias**
  - **No explicit mention**; study conducted in a single UK setting without comparative cultural analysis.
- **Limited focus on cost-of-caring mechanisms**
  - **No explicit discussion** of moral distress, compassion fatigue, vicarious trauma, or burnout mechanisms.

APA reference

- Nirodi, P., El-Sayeh, H., & Henfrey, H. (2018). Applying the apprenticeship model to psychiatry: An evaluation. *Progress in Neurology and Psychiatry, 22*(1), 25–29.

Sources

23. Pre-registration house...
PDF

Please analyse the following article and provide relevant verbatim statements that support these points. If there are no relevant mention of the points, please say so: 1) Studies described programs as facilitating internalization of professional values and norms through participation in clinical teams. 2) Workplace identity development was reported as trainees assumed increasing responsibility. 3) Studies identified several limitations of apprenticeship approaches beyond supervisor constraints. 4) Variability in program structure, duration, and components challenged standardization efforts.

Thought for 3m 36s

Article analysed

- Bleakley, A. (2002). *Pre-registration house officers and ward-based learning: A "new apprenticeship" model*. **Medical Education, 36**, 9–15. 23. Pre-registration house offi...

---

1) Programs facilitating internalization of professional values and norms through participation in clinical teams

- **Socialisation into professional values**
  - "Understanding the PRHO apprenticeship year should include reference to cultural dimensions to learning, especially socialisation into the profession" (Bleakley, 2002, p. 9). 23. Pre-registration house offi...
- **Values and norms learned beyond technical skills**
  - "Such psychological discussions of ward-based pedagogy miss the cultural process of socialisation into attitudes and values informing professional practice" (Bleakley, 2002, p. 10). 23. Pre-registration house offi...
- **Participation within clinical teams**
  - "PRHOs, as novices, are not relegated to passive learning roles, but may actively co-construct knowledge with experts" (Bleakley, 2002, p. 9).
    23. Pre-registration house offi...

- **Communities of practice**
  - "A socialisation into varying 'communities of practice' through work-based experience" (Bleakley, 2002, p. 9). 23. Pre-registration house offi...

---

2) Workplace identity development as trainees assumed increasing responsibility

- **Identity formation central to workplace learning**
  - "The PRHO year can be seen as a formal apprenticeship into the profession of medicine, and as central to the identity construction of the doctor" (Bleakley, 2002, p. 9). 23. Pre-registration house offi...
- **Identity constructed through participation rather than observation**
  - "Apprentices are not collecting learning but are in the process of constructing identities" (Bleakley, 2002, p. 12). 23. Pre-registration house offi...
- **Assumption of more active and responsible roles**
  - "The PRHO can play a significant role that moves well beyond the limits of 'novice' status" (Bleakley, 2002, p. 11). 23. Pre-registration house offi...
- **Progressive responsibility**
  - "Increased access to participating roles in expert performances" (Bleakley, 2002, p. 13). 23. Pre-registration house offi...

---

3) Limitations of apprenticeship approaches beyond supervisor constraints

- **Under-theorisation and limited evidence base**
  - "Teaching and learning in ward-based environments is under-researched" (Bleakley, 2002, p. 9). 23. Pre-registration house offi...
- **Limits of transmission-based apprenticeship models**
  - "Such a model offers a necessary, but not sufficient, explanation of how work-based learning occurs" (Bleakley, 2002, p. 9). 23. Pre-registration house offi...
- **Hierarchical and cultural barriers**
  - "Role concerns appeared to stifle, rather than facilitate, supportive educational dialogue" (Bleakley, 2002, p. 12). 23. Pre-registration house offi...
- **Restricted access to authentic practice**
  - "PRHOs … crave more bedside hands-on investigation of patients, but are often not given the opportunity" (Bleakley, 2002, p. 13). 23. Pre-registration house offi...
- **Structural trade-offs**

- "Longer PRHO attachments … would necessarily restrict wider experience of specialties" (Bleakley, 2002, p. 13). 23. Pre-registration house offi...

- **Limits to transferability of learning**
  - "Hope for the smooth transfer of generic learning across the cultural divides of firms may be misplaced" (Bleakley, 2002, p. 13). 23. Pre-registration house offi...

---

4) Variability in program structure, duration, and components challenging standardization efforts

- **Marked variability in ward-based contexts**
  - "Ward cultures differ considerably, as do the styles and habits of consultants' teaching, even within the same specialties" (Bleakley, 2002, p. 9). 23. Pre-registration house offi...
- **Limits to uniform educational models**
  - "We cannot assume an invariant process of ward-based teaching and learning" (Bleakley, 2002, p. 14). 23. Pre-registration house offi...
  - "There is no generic pedagogic formula" (Bleakley, 2002, p. 14). 23. Pre-registration house offi...
- **Critique of standardisation**
  - "An expectation for a uniform method of teaching and learning in ward round contexts" is explicitly problematised (Bleakley, 2002, p. 9). 23. Pre-registration house offi...

APA reference

- Bleakley, A. (2002). Pre-registration house officers and ward-based learning: A "new apprenticeship" model. *Medical Education, 36*(1), 9–15.

Sources
