# Supplementary material for: The role of physician apprenticeship models in the cost of caring: an AI-assisted systematic scoping review
Source: BMC Med Educ. 2026 Jul 27;26:1706. doi: 10.1186/s12909-026-09932-y (PMC13692915; doi:10.1186/s12909-026-09932-y)
Supplement: Supplementary file 3 [file 12909_2026_9932_MOESM3_ESM.docx]

# **Appendix S2. Search Strategy**

**Total numbers:**

| **Database/AI Platform** | **Number of results** | |
| --- | --- | --- |
|  | **Without year filter** | **With year filter** |
| PubMed | 3,563 | 3,045 |
| Embase | 1,665 | 1,626 |
| ERIC | 343 | 224 |
| Scopus | 1726 | 1,696 |
| Google Scholar | 685 | 651 |
| Elicit AI | 199 | 92 |
| **Total** | 8,181 | 7,334 |

# **PubMed**

**Date conducted:**

24/1/2026

## Full search strategy:

("Clinical Clerkship"[Mesh] OR "Preceptorship"[Mesh] OR "clinical clerkship*"[tiab] OR "clerkship*"[tiab] OR "apprentice*"[tiab] OR "preceptor*"[tiab]) AND ("Professionalism"[Mesh] OR "Social Identification"[Mesh] OR "Professional Role"[MeSH] OR "Social Values"[Mesh] OR "Professional competence"[Mesh] OR "Self-concept"[Mesh] OR "professional identity"[tiab] OR "professional identities"[tiab] OR "professionalism*"[tiab] OR "e-professionalism*"[tiab] OR "identity formation*"[tiab] OR "social identification"[tiab] OR "socialization"[tiab] OR "socialisation"[tiab] OR "professional role"[tiab] OR "professional roles"[tiab] OR "social value*"[tiab] OR "professional competence"[tiab] OR "self-concept"[tiab]) AND ("Medicine"[Mesh] OR "Education, Medical"[Mesh] OR "Schools, Medical"[Mesh] OR "Students, Medical"[Mesh] OR "Internship and residency"[Mesh] OR "Physicians"[Mesh] OR "medical student*"[tiab] OR "medical school*"[tiab] OR "medicine"[tiab] OR "doctor*"[tiab] OR "physician*"[tiab] OR "trainee*"[tiab] OR "resident"[tiab] OR "residents"[tiab])

("Clinical Clerkship"[Mesh] OR "Preceptorship"[Mesh] OR "clinical clerkship*"[tiab] OR "clerkship*"[tiab] OR "apprentice*"[tiab] OR "preceptor*"[tiab])

AND

("Professionalism"[Mesh] OR "Social Identification"[Mesh] OR "Professional Role"[MeSH] OR "Social Values"[Mesh] OR "Professional competence"[Mesh] OR "Self-concept"[Mesh] OR "professional identity"[tiab] OR "professional identities"[tiab] OR "professionalism*"[tiab] OR "e-professionalism*"[tiab] OR "identity formation*"[tiab] OR "social identification"[tiab] OR "socialization"[tiab] OR "socialisation"[tiab] OR "professional role"[tiab] OR "professional roles"[tiab] OR "social value*"[tiab] OR "professional competence"[tiab] OR "self-concept"[tiab])

AND

("Medicine"[Mesh] OR "Education, Medical"[Mesh] OR "Schools, Medical"[Mesh] OR "Students, Medical"[Mesh] OR "Internship and residency"[Mesh] OR "Physicians"[Mesh] OR "medical student*"[tiab] OR "medical school*"[tiab] OR "medicine"[tiab] OR "doctor*"[tiab] OR "physician*"[tiab] OR "trainee*"[tiab] OR "resident"[tiab] OR "residents"[tiab])

# **Embase**

**Date conducted:**

15/1/2026

## Full search strategy:

('clinical education'/exp OR 'clerkship'/exp OR 'apprenticeship'/exp OR 'clinical clerkship*':ti,ab OR clerkship*:ti,ab OR apprentice*:ti,ab OR preceptorship*:ti,ab) AND ('professionalism'/exp OR 'social identity'/exp OR 'social value'/exp OR 'professional competence'/exp OR 'self concept'/exp OR 'professional identit*':ti,ab OR professionalism*:ti,ab OR 'e-professionalism*':ti,ab OR 'identity formation*':ti,ab OR 'social identification':ti,ab OR socialization:ti,ab OR socialisation:ti,ab OR 'professional role':ti,ab OR 'professional roles':ti,ab OR 'social value*':ti,ab OR 'professional competence':ti,ab OR 'self-concept':ti,ab) AND ('medicine'/exp OR 'medical education'/exp OR 'medical school'/exp OR 'medical student'/exp OR 'internship'/exp OR 'residency'/exp OR 'physician'/exp OR 'medical student':ti,ab OR 'medical school*':ti,ab OR 'medicine':ti,ab OR 'doctor*':ti,ab OR 'physician*':ti,ab OR 'trainee*':ti,ab OR 'resident':ti,ab OR 'residents':ti,ab) AND [2000-2023]/py

'clinical education'/exp OR 'clerkship'/exp OR 'apprenticeship'/exp OR 'clinical clerkship*':ti,ab OR clerkship*:ti,ab OR apprentice*:ti,ab OR preceptorship*:ti,ab

AND

'professionalism'/exp OR 'social identity'/exp OR 'social value'/exp OR 'professional competence'/exp OR 'self concept'/exp OR 'professional identit*':ti,ab OR professionalism*:ti,ab OR 'e-professionalism*':ti,ab OR 'identity formation*':ti,ab OR 'social identification':ti,ab OR socialization:ti,ab OR socialisation:ti,ab OR 'professional role':ti,ab OR 'professional roles':ti,ab OR 'social value*':ti,ab OR 'professional competence':ti,ab OR 'self-concept':ti,ab

AND

'medicine'/exp OR 'medical education'/exp OR 'medical school'/exp OR 'medical student'/exp OR ‘internship’/exp OR ‘residency’/exp OR 'physician'/exp OR 'medical student':ti,ab OR 'medical school*':ti,ab OR 'medicine':ti,ab OR 'doctor*':ti,ab OR 'physician*':ti,ab OR 'trainee*':ti,ab OR 'resident':ti,ab OR 'residents':ti,ab

# **ERIC**

**Date conducted:**

20/1/2026

## Full search strategy:

(MAINSUBJECT.EXACT.EXPLODE("Apprenticeships") OR MAINSUBJECT.EXACT.EXPLODE("Clinical Teaching (Health Professions)") OR MAINSUBJECT.EXACT.EXPLODE("Clinical Experience") OR MAINSUBJECT.EXACT.EXPLODE("Practicum Supervision") OR (TI,AB(("clinical clerkship" OR "clinical clerkships") OR clerkship* OR apprentice* OR preceptor*))) AND (MAINSUBJECT.EXACT.EXPLODE("Professionalism") OR MAINSUBJECT.EXACT.EXPLODE("Social Values") OR MAINSUBJECT.EXACT.EXPLODE("Professional Identity") OR MAINSUBJECT.EXACT.EXPLODE("Self Concept") OR MAINSUBJECT.EXACT.EXPLODE("Professional Development") OR (TI,AB("professional identity" OR "professional identities" OR professionalism* OR "e-professionalism*" OR ("identity formation") OR "social identification" OR socialization OR socialisation OR "professional role" OR "professional roles" OR ("social value" OR "social values") OR "professional competence" OR "self-concept"))) AND (MAINSUBJECT.EXACT.EXPLODE("Medicine") OR MAINSUBJECT.EXACT.EXPLODE("Medical Education") OR MAINSUBJECT.EXACT.EXPLODE("Medical Schools") OR MAINSUBJECT.EXACT.EXPLODE("Medical Students") OR MAINSUBJECT.EXACT.EXPLODE("Graduate Medical Education") OR MAINSUBJECT.EXACT.EXPLODE("Physicians") OR (TI,AB(("medical student" OR "medical students") OR ("medical school" OR "medical schools") OR medicine OR doctor* OR physician* OR trainee* OR resident OR residents)))

(MAINSUBJECT.EXACT.EXPLODE("Apprenticeships") OR MAINSUBJECT.EXACT.EXPLODE("Clinical Teaching (Health Professions)") OR MAINSUBJECT.EXACT.EXPLODE("Clinical Experience") OR MAINSUBJECT.EXACT.EXPLODE("Practicum Supervision") OR (TI,AB(("clinical clerkship" OR "clinical clerkships") OR clerkship* OR apprentice* OR preceptor*)))

AND

(MAINSUBJECT.EXACT.EXPLODE("Professionalism") OR MAINSUBJECT.EXACT.EXPLODE("Social Values") OR MAINSUBJECT.EXACT.EXPLODE("Professional Identity") OR MAINSUBJECT.EXACT.EXPLODE("Self Concept") OR MAINSUBJECT.EXACT.EXPLODE("Professional Development") OR (TI,AB("professional identity" OR "professional identities" OR professionalism* OR "e-professionalism*" OR ("identity formation") OR "social identification" OR socialization OR socialisation OR "professional role" OR "professional roles" OR ("social value" OR "social values") OR "professional competence" OR "self-concept")))

AND

(MAINSUBJECT.EXACT.EXPLODE("Medicine") OR MAINSUBJECT.EXACT.EXPLODE("Medical Education") OR MAINSUBJECT.EXACT.EXPLODE("Medical Schools") OR MAINSUBJECT.EXACT.EXPLODE("Medical Students") OR MAINSUBJECT.EXACT.EXPLODE("Graduate Medical Education") OR MAINSUBJECT.EXACT.EXPLODE("Physicians") OR (TI,AB(("medical student" OR "medical students") OR ("medical school" OR "medical schools") OR medicine OR doctor* OR physician* OR trainee* OR resident OR residents)))

# **Scopus**

**Date conducted:**

20/1/2026

## Full search strategy:

TITLE-ABS-KEY ( *clinical AND clerkship OR *clerkship OR *apprentice OR *preceptor ) AND ( *professional AND identity OR *professionalism* OR *e-professionalism OR *identity AND formation OR *socialization OR *socialisation OR *professional AND role OR *professional AND roles OR *social AND value ) AND ( *medical AND student OR *medical AND school OR *medicine OR *doctor OR *physician OR *trainee OR *resident OR *residents ) AND PUBYEAR > 2000 AND PUBYEAR < 2024

("clinical clerkship*" OR clerkship* OR apprentice* OR preceptor*)

AND

("professional identit*" OR professionalism* OR "e-professionalism*" OR "identity formation*" OR socialization OR socialisation OR "professional role" OR "professional roles" OR "social value*")

AND

("medical student*" OR "medical school*" OR medicine OR doctor* OR physician* OR trainee* OR resident OR residents)

# **Google Scholar**

**Date conducted:**

16/1/2026

## Full search strategy:

With all of the words: Medical medicine apprenticeship doctor

With the exact phrase: Professional Identity Formation

With at least one of the words: student resident trainee physician

Without the words: lawyer legal law "pre-service" preservice engineer

Where my words occur: Anywhere in the article

**Elicit AI**

**Date conducted:**
20/1/2026

**Full search strategy**

Research Question: (“Could apprenticeship offer a means of better supporting physicians confronting the cost of caring?”*) AND PUB > 1 January 2000 AND < 30 June 2025

| Prompt | ("apprenticeship in medicine published in 2025*”)  AND |
| --- | --- |
| Concept | ("Apprenticeship in medical education, its effect on PIF, Professional Identity Formation, and similarities with mentoring*")  AND |
| Context | ("Medical Education*”)  AND |
| Population Exclusion | ("Does it exclude: ● Allied health specialties, such as dietetics, nursing, psychology, chiropractic, midwifery, social work ● Non-medical specialties, such as clinical and translational science, veterinary, dentistry*”)  AND |
| Population Inclusion | ("Does it include: ● Junior physicians, house officers, postgraduate year one physicians ● Medical officers, senior medical officers, registrars, specialist registrars ● Residents, senior residents, clinical fellows ● Undergraduate and postgraduate medical students”) |
