# Supplementary material for: The role of physician apprenticeship models in the cost of caring: an AI-assisted systematic scoping review
Source: BMC Med Educ. 2026 Jul 27;26:1706. doi: 10.1186/s12909-026-09932-y (PMC13692915; doi:10.1186/s12909-026-09932-y)
Supplement: Supplementary file 4 [file 12909_2026_9932_MOESM4_ESM.docx]

**Appendix S3. Summary of Included Articles**

| Citation number | Author, Year | Article title | Type of study | Study aim and methodology | Key findings | Insights/conclusions | MERSQI/COREQ |
| --- | --- | --- | --- | --- | --- | --- | --- |
| 50 | Boudreau et al., 2014 | Affirming Professional  Identities Through an Apprenticeship: Insights From a Four-Year Longitudinal Case Study | Mixed methods | The goal of this study was to understand the apprenticeship learning process, particularly its contribution to professional identity formation. | Physician Apprenticeship activities promoted and sustained medical  professionalization in the participants. Salient features of successful apprenticeship learning were access to authentic clinical experiences as well as the provision of a safe learning environment and guided critical reflection. The latter two ingredients appear to be mutually reinforcing and contributed to the creation of meaningful student–teacher relationships. Teachers exhibited several qualities that align with a parental role.  Students became increasingly aware of having entered the kinship of physicians. Teachers experienced a renewal and validation of their commitment to the ideals of medicine. | Findings strongly suggest that a longitudinal apprenticeship in an undergraduate medical program can contribute to the formation and reaffirmation of professional identity. The case study design permitted the authors to create a provisional conceptual model explicating important features of the apprenticeship learning process. | 10 |
| 51 | Balmer et al.,  2008 | Understanding paediatric resident-continuity preceptor relationships through the lens of apprenticeship learning | Qualitative | We conducted an ethnographic case study of paediatric residents learning relationships with their preceptors in a community-based paediatric continuity site. It included 5 months (100 hours) of direct observation, and semi-structured interviews with 10 residents (before and after observation) and 10 primary care paediatricians who served as their continuity preceptors (after observation). | Our observations and reports of resident learning trajectories fit well with the concept of legitimate peripheral participation. Residents learned the everyday practice of primary care as they worked alongside experienced paediatricians in the continuity clinic. Although the direction of learning was towards central participation in patient care, residents learned during transient shifts to the periphery of practice. As a function of residents increased participation, preceptors moved into more supportive roles. Residents were not only learners; at times they were teachers who facilitated preceptors learning. | Legitimate peripheral participation is a concept that helps to explain apprenticeship as a dynamic social relationship which shapes, and is shaped by, learning that takes place in clinical practice. Other concepts shed light on the bidirectional nature of apprenticeship learning. | 48 |
| 52 | Braniff et al.,  2015 | Assistantship improves medical students’ perception of their preparedness for starting work | Quantitative | The aim of the study was to assess if medical students felt that the assistantship improved their preparedness for starting work as FY1s. | There was a statistically significant improvement in the students’ perception of their preparation for 49 of 56 tasks contained within the questionnaire. After the assistantship 81.2% of students felt well prepared for starting work compared with 38.9% before the assistantship.  93.9% agreed that the assistantship had improved their preparedness for starting work. | The assistantship at Queen’s University improves medical students’ perception of their preparedness for starting work. The majority of medical students feel well prepared for starting work after completing the assistantship. | 11 |
| 53 | Ryan et al.,  2017 | Preparation for practice: a novel role for general practice in pre-foundation assistantships | Qualitative | We sought to explore what spending one week of the pre-foundation assistantship in General Practice might add.  We solicited reflective audio diaries from final  year students during a one-week pilot attachment delivered during the post-finals, prefoundation assistantship period, and performed an iterative thematic analysis on the acquired data. | From this attachment in General Practice, students described diverse learning, resulting in improved preparedness for (hospital) foundation practice across several domains, impacting positively on how they might approach patients in the future. Self-confidence improved due to affirming outcomes and tutor mentorship. Students deepened their understanding of community healthcare and General Practice; and seeing the ‘Patient Journey’ across the interface from the patient’s perspective helped them  contextualise their forthcoming role as foundation doctors in managing it. | We believe that this novel intervention distinctively contributed to  preparedness for practice. It aligns with published recommendations about extending the current assistantship model. We suggest it should be incorporated more widely into prefoundation assistantship curricula. | NA |
| 54 | Cloyd et al.,  2008 | Operating Room Assist:  Surgical Mentorship and  Operating Room  Experience for  Preclerkship Medical  Students | Mixed | In conjunction with a previously described surgical skills elective, we created a new clinical elective in which preclerkship medical students were assigned a surgical mentor and invited into the operating room to assist in surgeries. The elective paired 36 first-year students with 24 surgeons and instructed students to participate in at least 2 surgeries over the 3-month elective period. Students, surgeons, and operating room nurses filled out questionnaires after each surgery. | Although 6 students failed to enter the operating room, 30 students scrubbed and gowned for a total of 62 procedures during the elective period. Although most students reported the operating room to be a comfortable learning environment in which they were actively included, students consistently underrated their performance and contribution to the surgical team compared with the surgeons' and nurses' ratings. With 75% of students who reported using a previously learned surgical skill during each surgery, this elective succeeded in allowing preclerkship medical students the opportunity to participate actively in the operating room. | Early surgical exposure is critical for attracting student interest in careers in surgery. We believe that these early clinical experiences, combined with strong mentorship from the surgical faculty, will eventually lead to greater success during clerkships and greater interest in surgery as a career. | 9.5 |
| 55 | Brown et al.,  2020 | Becoming a clinician: Trainee identity formation within the general practice supervisory relationship | Qualitative | Our aim was to develop a conceptualisation of trainee identity formation within the general practice (GP) supervisory relationship to aid its support. | We identified three social Discourses centring on: clinical responsibility; ownership of clinical knowledge; and, measures of trainee competency. Versions of these Discourses defined four trainee/supervisor relational arrangements within which trainee and supervisor assumed reciprocal identities. | In the supervisory relationship, trainee and supervisor adopted reciprocal identities. For trainees to progress to identity of lead clinician, supervisors needed to invite their trainee into this space and vacate it themselves. Congruence between supervisor positioning of their trainee and trainee authorship of themselves was important and was aided by explicit dialogue and common purpose. We offer a model and language for trainees, supervisors and faculty to facilitate this. | 6-14 |
| 56 | Morris et al.,  2015 | Evaluation of a “Subintern”  Role: Action Over  Observation For Final-Year Medical Students in  Surgery | Mixed methods | This study sought to investigate the potential of a subinternship apprentice role as a structured experiential learning opportunity by enabling final-year medical students to learn through action rather than observation. | Wilcoxon rank sum test results showed that students participating in the “subintern” group had a  statistically significantly better clinical experience than the “intern-shadow” group regarding role modeling (p 1⁄4 0.0002), learning and teaching (p 1⁄4  0.0021), organization and support (p 1⁄4 0.0219), and patient interaction (p 1⁄4 0.0063). | The “subintern” apprentice role shows strong potential for enabling final-year students to learn on the job by actually performing the intern role rather than merely observing an intern at work. Subinterns reported feeling valued within the team structure and expressed a desire to emulate expert practice directly experienced through close collaboration with consultant surgeons. | MERSQI: 13.5 |
| 57 | O’Doherty et al.,  2021 | Medical students and clinical placements - a qualitative study of the continuum of professional identity formation | Qualitative | To explore graduates’ perceptions of significant factors affecting professional identity formation (PIF) throughout their graduate medical school education journey and early practice years. A qualitative study with medical graduates using non-probability sampling. Data collected with graduates via face to face and telephone interviews. Interviews (n = 9) completed with medical graduates of the School of Medicine, University of Limerick. | Graduates described their experiences in general practice, during the early patient contact programme and the longitudinal integrated clerkship (LIC) as highly influential. The lasting impact of positive role models was highlighted. The importance of socialisation and entering a community of practice were identified as drivers of professional development. Role modelling and mentorship between students and GP tutors were pivotal as part of early clinical years and clinical LIC. This seemed to have a positive influence on graduate’s consideration of general practice as a future career pathway. | Professional identity formation occurs for medical students who participate in early patient contact programmes and longitudinal integrated clerkships in GP. Factors such as positive role modelling, good mentorship, communities of practice and a positive learning environment appear to be the main contributors to this process. Experiences as part of longitudinal integrated clerkships are meaningful for graduates, regardless of postgraduate specialisation choices. Educators should acknowledge this when designing medical curricula to ensure that students’ professional identity formation is optimally facilitated. Training should be available to support the educators involved in longitudinal integrated clerkships, as they become role models and mentors to students. | COREQ: 1-5 |
| 58 | Kenny et al.,  2003 | Role Modeling in  Physicians’ Professional Formation: Reconsidering an Essential but Untapped Educational Strategy | Descriptive | This article identifies some foundational questions regarding role models and professional character formation, describes the major social and historical reasons for inattention to the character formation of new physicians, draws insights from disciplines outside medical education that can inform our understanding of this important area, and suggests the practical consequences of this work for faculty recruitment, affirmation, and development. | We need to: clarify the foundational definitions of “role,” “models,” and “role modeling,”, identify core attributes of good clinical practice and those that are specialty specific, address faculty recruitment and development as role models, involve  faculty in an analysis of their environment, develop strategies whereby faculty can reflect on their own experience and learn from it, assist faculty with adapting their teaching and modeling accordingly, assist learners to develop strategies for identifying good and poor role modeling and conscious reflective maneuvers to deal with the potential influence of these models, develop safe spaces where negative role modeling can be reflected upon and  translated into an effective learning experience | Role modeling is at the heart of professional character formation. Excellence in professional practice is learned in  and through experience and critical reflection on its expression in the clinical encounter. Knowledge and skills are  essential, but putting them together in a competent and caring response to patients’ needs is learned in personal interaction and role modeling. We must focus our attention  more critically on this fundamental aspect of physicians’ professional character formation for the benefit of both faculty and learners. | NA |
| 59 | Nishikura N., et al. (2021) | Effect of Residents-as-Teachers in Rural Community-Based  Medical Education on the Learning of Medical Students  and Residents: A Thematic Analysis | Thematic analysis | Residents-as-teachers (RaT) is a theoretical framework emphasizing the significance of the  similar learning background of teachers and learners. In Japan, community-based medical education  (CBME) is a practical approach to teaching family medicine. This study aimed to investigate the  impact and challenges of RaT on the learning of medical students and residents in CBME at a rural  community hospital in Japan. | Over the course of a year, the researchers conducted one-on-one  interviews with three residents and ten medical students participating in family medicine training at  the hospital. The interviews were recorded and transcribed verbatim. Grounded theory was used  in the data analysis to clarify the findings. | Three key themes emerged from the research: lack of  educational experience with RaT, effectiveness of RaT, and challenges of RaT. Although participants  were prejudiced against RaT, they felt its implementation could facilitate the establishment of beneficial  relationships between learners and teachers. They were also able to participate in medical  teams effectively. | NA |
| 60 | Nirodi et al.,  2018 | Applying the  apprenticeship model to psychiatry: an evaluation | Mixed methods | This article assesses the success of an apprenticeship scheme piloted within a mental health setting for fourth year medical students. | The results show that it was possible and indeed successful in terms of fulfilling the basic tenets of the apprenticeship model. It was also found to be a novel method of increasing the likelihood of students considering psychiatry as a future career. | The results of this preliminary study into the applicability of an  apprenticeship scheme in psychiatry suggest that this is possible and indeed successful in terms of fulfilling the basic tenets of an apprenticeship. | COREQ: 25-28 MERSQI: 6 |
| 61 | Bleakley, 2002 | Pre-registration house officers and ward-based  learning: a 'new apprenticeship' model | Discussion paper | Teaching and learning in ward-based environments is under-researched, and the current literature displays a bias towards a psychological model of pedagogy that focuses upon transmission of knowledge and skills from one individual to another. Such a model offers a necessary, but not sufficient, explanation of how work-based learning occurs. Understanding the PRHO apprenticeship year should include reference to cultural dimensions to learning, especially socialisation into the profession. This constitutes an 'extended' (or 'hidden') curriculum  model that may be theorised through contemporary ideas of activity learning within a 'new apprenticeship' framework. | The dominant psychological model can lead to an expectation for a uniform method of teaching and learning in ward round contexts that (a) ignores important differences in educational climate between established communities of practice, and (b) orients both teachers and learners to one-to-one transmission and reception, rather than sensitising to how knowledge may be held across members of a working group. The latter shifts emphasis away from reception to issues of active access. PRHOs, as novices, are not relegated to passive learning roles, but may actively co-construct knowledge with experts, offering potential transformation of the practices of ward groups. | A new model of apprenticeship, framing  learning as a cultural activity of participation embedded in the possibility of transformation of the culture itself, suggests possible educative interventions that may considerably enhance the value of PRHO ward-based attachments. Importantly, in terms of the possible transformation of a working group's established practices, the PRHO can play an active part, despite ‘novice’ status. | NA |
| 62 | Jones et al.,  2004 | An Intentional Modeling Process to Teach  Professional Behavior:  Students’ Clinical  Observations of  Preceptors | Mixed methods | The authors discuss an innovative process, called Students’ Clinical Observations of Preceptors (SCOOP), which reverses the traditional direction of struc- tured observations. With written cues to focus their observations, students observe their preceptors, who intentionally model professionalism and communication during clinical encounters. Students and preceptors discuss the observed patient–physician interaction during post-encounter sessions. | Most medical students rated the SCOOP process highly and reported professional behaviors they gained. | As educators seek methods for learners to attain greater competence in communication and interpersonal skills, the SCOOP provides an explicit framework to optimize modeling for the learning of professionalism. | MERSQI: 12    COREQ:  264, 266-268 |
| 63 | Balmer et al.,  2007 | Learning Behind the Scenes: Perceptions and  Observations of Role Modeling in Pediatric Residents’ Continuity  Experience | Qualitative | Little is known about what residents learn from “everyday” physician role models, who, in the course of their ordinary work, serve as real-life examples of residents’ future roles. The purpose of this research was to analyze what and how pediatric residents learn through role modeling during their con-tinuity experience.  We conducted a case study of 10 third-year pediatric residents and their 10 continuity clinic preceptors (CCP) in a community-based continuity clinic. Data were derived from 5 months (100 hours) of direct observation in clinic; semistruc- tured interviews with residents before and after observation; and semi-structured interviews with CCPs after resident data were collected. Interview transcripts and notes from observation were inductively coded and thematically analyzed. | From the residents’ perspective, role modeling was an implicit and intentional learning strategy that was linked to routine clinical practice in continuity clinic. Residents learned, through modeling their CCPs, “how to talk” and “how to think things through.” Residents did not directly report modeling pro- fessional behavior. For residents, learning through modeling was not contingent on CCPs’ awareness of being a role model. | Role modeling is a nuanced, deliberate learning strategy that provides pediatric residents with templates for in- terpersonal communication and clinical decision making that have both immediate and long-term relevance. Understanding residents’ perspective on role modeling, and how it aligns with their CCPs’ perspective, presents opportunities for improving residents’ learning experiences, faculty development, and future research. | COREQ: 176-180 |
| 64 | Cruess, R. L., et al. (2014) | Reframing medical education to support professional identity formation | Review | Teaching medical professionalism is a fundamental component of medical education. The objective is to ensure that students understand the nature of professionalism and its obligations and internalize the value system of the medical profession. The recent emergence of interest in the medical literature on professional identity formation gives reason to reexamine this objective. | NA | NA | NA |
| 65 | Osterberg et al.,  2016 | Back to the Future: What  Learning Communities  Offer to Medical Education | Descriptive | The purpose of this study is to provide a rationale for learning communities (LCs) as a paradigm in medical education and describe many benefits LCs have in supporting the professional identity formation of medical students. | Not indicated | LCs play a critical role in medical students’ professional identity formation with the social and relational roles that LC faculty play using active role modeling, promoting reflection, longitudinal mentoring, and helping students reconcile the challenges they face in medical schools’ formal and hidden curriculum. If LCs are carefully structured, they can help break down the exclusionary nature of the traditional medical profession by systematically providing all students with a faculty mentor and a small group of near peers with whom they can reflect as they navigate the challenges of medical school. The structure of LCs gives inherent support to students by incorporating explicit wellness functions, promoting a supportive environment for all students, and providing an important safety net given the high-risk medical students have for isolation, depression, and burnout.  Given the important role patients play in the personal and professional development of medical trainees, a future consideration in LCs would be to broaden the continuity model to include patient-student-faculty in future LCs. | NA |
| 66 | Henschen et al., 2015 | Four-Year Educational and Patient Care Outcomes of a Team-Based Primary Care Longitudinal Clerkship | Quantitative | All students graduating in 2015 were surveyed to assess attitudes, experiences, and preferences regarding primary care education. ECMH students were compared with students receiving  their primary care training in a traditional curriculum (TC) using paired measures of comparison. To assess the impact of the ECMH on patient care quality, authors performed a detailed chart review at one site. | ECMH students reported more continuity-of-care experiences, higher satisfaction with their primary care learning climate (86% versus 61% in the EMCH and TC cohorts, respectively), more confidence in their quality improvement skills, and scored higher on measures of perceived patient centeredness. Students from both groups recommended the ECMH (91% and  57%, respectively). Student involvement at one ECMH site was correlated with increased patient contacts and improved delivery of recommended preventive care. | Incorporating students longitudinally into primary care clinics is highly rated by students. The ECMH model led to improved continuity, improved perceptions of the learning climate, and higher patient centeredness.  Preliminary data suggest that students add value and improve patient outcomes during longitudinal clinical experiences. | 15 |
| 67 | Bettin, 2021 | The Role of Mentoring in the Professional Identity  Formation of Medical  Students | Review Paper | This review specifically defines PIF for medical students, discussing factors that influence PIF, exploring the role of mentoring on the PIF of medical students, and suggesting ways to use mentoring and PIF to increase interest of medical students, especially women and underrepresented minority students, in the field of orthopedics. | Professional identity formation is a lifelong, iterative process whereby a medical student develops a sense of self and embodies the ideals and values of a physician.    Socialization is key for the formation of professional identity for medical students, including formal and informal curriculum, clinical experiences, role modeling, and mentorship and narrative reflection.    Of these, positive mentoring relationships with experienced faculty members has been shown to improve students’ satisfaction with their career choices, boost confidence in career choices, and give them an opportunity to form their professional identity in a safe, nurturing environment. | Physician educators and mentors can offer personal guidance, academic support, and the ability for students to reflect on the components of their personal identity and how this personal identity informs and contributes to their professional identity.    Mentorship is  a way continue the ideals, values, skills, culture, research, and innovation in a specialty and helps ensure that a new generation of diverse trainees understands the gravity and embodies the depth of what it means to be an orthopedic surgeon. | NA |
| 68 | Lyons et al.  2016 | Cognitive apprenticeship in health sciences education: a qualitative review | Qualitative | A growing body of literature provides empirical support for the theory of cognitive apprenticeship and suggests that health sciences educators are incorporating this frame- work into their practices in various ways. Currently, the cognitive apprenticeship literature is dispersed across various disciplines, including nursing, medicine, pharmacy, and veterinary medicine, and applied within various settings, including classrooms, clinics, and online environments. The purpose of this qualitative review was to synthesize and evaluate the use of cognitive apprenticeship theory in health sciences education research with the primary aim of understanding how and to what extent the theory of cognitive apprenticeship has been applied to education in health sciences. By synthesizing the use of cognitive apprenticeship in health sciences education, this work can extend our under- standing of theories that underpin educational practice and inform pedagogy and research.  The search strategy was shaped by the goals of this qualitative review: to describe and evaluate the use of cognitive apprenticeship as a theoretical model in published health sciences education research. We conducted preliminary searches in health sciences and education databases (PubMed, CINAHL, PsychInfo and Education Full Text) using terms ‘‘cognitive apprenticeship’’ and some related terms, such as  ‘‘scaffolding,’’ ‘‘situated cognition,’’ and  ‘‘reflection,’’ as well as various combinations of those terms with ‘‘cognitive apprenticeship.’’ | Articles using major theory talk (n =  26) are listed in Appendix Table 5. All  26 articles were published between 1990 and 2014, with the majority published in the last 5 years (n = 18). Six countries were represented, with most articles originating from the Netherlands (n = 8), United States (n = 7), and United Kingdom (n = 6). The majority of the articles were focused on medical education (n = 14), with ten articles about medical residents and practitioners and four about medical students. The remaining articles focused on nursing or midwife education (n = 7), veterinary education (n = 3), dental education (n = 1), and pharmacy education (n = 1). The results of this review are structured by question to summarize relevant findings. | Cognitive apprenticeship theory provides a framework for developing expertise in health sciences students. As seen in these studies, a number of researchers have successfully translated cognitive apprenticeship principles to health science learning environments in an effort to prepare students for the rapidly changing healthcare needs of society. Expanding our application of cognitive apprenticeship and providing faculty development in this area may further improve development of professional expertise. New research directions should apply the theory into additional aspects of health sciences educational research, including classroom learning and interprofessional education. | Systematic review  MERSQ NA COREQ NA |
| 69 | Wearne et al., 2016 | Educating registrars in your practice | Editorial | This article summarises how registrars learn, and the role of general practice supervisors and training practices in supporting their education. | Descriptive in nature, no new findings/results were forwarded | In summary, the apprenticeship model of learning how to be a GP by working as a GP is fundamental. One supervisor needs to take overall responsibility for planning and supporting learning within the practice, and for proactive and reactive supervision | NA |
| 70 | Peer, 2016 | Professional Identity  Formation: Considerations for Athletic Training  Education | Descriptive | Although many clinical education models have evolved over the decades, most of these models lack emphasis on the integral role of identity formation. Newer models contend that a clinical immersion experience fostering the optimal mix of challenge and support facilitates identity formation. | First, identity is a negotiated experience that is defined not only by how students define themselves, but also by how others see them. Next, identity within a community is established by interpretation of familiar and unfamiliar cues encountered in the learning environment. Identity is also a factor of our self-defined learning trajectory, integrating where we have been with where we want to be in the future. Fourth, identity is formed through the reconciliation of the multiple relationships into one emergent identity. Lastly, personal professional identity is formulated through the convergence of becoming a member of proximal groups as well as broader groups such as the profession as a whole. | Concerted efforts to embrace the various theories and principles related to collaborative and socially constructed engagement in the educational process should be taken so that the transition to the master’s program yields confident, self-regulated practitioners who fully understand their role and possess a strong professional identity within the interprofessional health care market. | NA |
| 71 | Thomas et al., 2021 | Teaching on Rounds and in Small Groups | Review paper | Not indicated | Not indicated | Not indicated | NA |
| 72 | Hay et al., 2013 | Medical students’ reactions to an  experience-based  learning model of clinical education | Qualitative | The purpose of the study was to check how the model, which is the product of a series of earlier research studies, aligned with students’ experiences, akin to the ‘member checking’ stage of a qualitative research project. In three group discussions, a researcher explained experience-based learning model (ExBL) to 19 junior clinical students, who discussed how it corresponded with their experiences of clinical learning and were given a written pre´cis of it to take away. One to 3 weeks later, they wrote 500word reflective pieces relating to their subsequent experiences with ExBL. | Having found many instances of responses ‘resonating’ to the model, the authors systematically identified and coded respondents’ ‘resonances’ to define how they aligned with their experiences. 120 resonances were identified. Seventy (58 %) were positive experiences and 50 (42 %) negative ones. Salient experiences were triggered by the learning environment in 115 instances (96 %) and by learners themselves in 5 instances (4 %), consistent with a strong effect of environment on learning processes. Affective support was apparent in 129 of 203 statements (64 %) of resonances and 118 learning outcomes (58 %) were also affective. | ExBL aligns with medical students’ experiences of clinical learning. Subject to further research, these findings suggest ExBL could be used to support the preparation of faculty and students for workplace learning | 14-66 |
| 73 | Lightman et al.,  2015 | A prolonged assistantship for final-year students | Mixed methods | This mixed methodology study explores the transition from medical student to junior doctor from the perspective of final- year medical students.  Final- year medical students responded to a questionnaire about anxieties surrounding their forthcoming foundation year 1 (FY 1). Students were purposefully sampled and semi- structured interviews (SSI s) were conducted, with 20 participants exploring their experience of the assistantship. Interviews were transcribed verbatim and thematic analysis was carried out. | The questionnaire results highlighted that most students felt anxious about becoming a FY 1 doctor, and subsequent interviews demonstrate that the assistantship mostly met with students’ expectations. Major themes regarding their anxieties included the value of building professional relationships, familiarisation with the  work environment, level of  supervision and the value of targeted teaching. | Student assistantships clearly improve preparedness and confidence in the transition to FY 1 by enabling supervised, stepwise responsibility. In the future a nationally cohesive programme should be created for shadowing and assistantships matched with students’ prospective jobs. | COREQ: 1-6 |
| 74 | Kittmer et al.,  2013 | Exploring the hidden curriculum: a qualitative analysis of clerks’ reflections on  professionalism in surgical clerkship | Qualitative | A small group discussion model using critical incident reports (CIRs) to elicit students’ reflections on ethical, communication, and professionalism challenges during surgical clerkship [was introduced]. We described the themes identified by surgical clerks in their CIRs.” “Using thematic analysis, 4 investigators coded 64 CIRs iteratively until conceptual saturation. Rigor and validity were ensured throughout the process. Data [was] further explored to compare the CIRs of junior and senior clerks. | Twenty-seven themes and 4 relationship domains emerged: the clerk’s relationship to patients, the health care team, the health care system, and self. Challenges with communication, the consent process, and breaking bad news were most commonly cited. Theme frequencies differed between junior and senior clerks. | Small group discussions of critical incident reports allow surgical clerks to reflect on their developing professional relationships. The themes that have been identified can be used to guide professionalism education and uncover the hidden curriculum. | 427-431 |
| 75 | Alford et al.,  2004 | Introducing First-Year Medical Students to  Clinical Practice by Having Them "Shadow" Third Year Clerks | Mixed methods | A new clinical experience was developed in which 1st-year medical students (MS1s) shadowed 3rd-year medical students (MS3s) as they performed their usual clerkship duties for 6 half-days, 1 in each clerkship. Researchers wanted to determine whether this was a worthwhile experience for MS1s and what they learned. MS1s documented their experiences shadowing MS3s in confidential responses to open-ended questions on a course Web site. Researchers conducted a qualitative analysis of student comments. | The analysis showed the students to be perceptive, thoughtful observers and yielded 5 broad categories of learning. Students learned about the practice of medicine, the process of becoming a doctor, providers of health care, the nature of real patients, and the procedures of medicine. Students rated the experience as “excellent.” | Shadowing an MS3 in clinical settings is a beneficial and valued learning experience for beginning medical students. | MERSQI: 10  COREQ: 260-263 |
| 76 | Iwata et al.,  2013 | Learning through work: Clinical shadowing of junior doctors by first year medical students | Mixed methods | To explore the potential of an ECC activity oriented to work as a junior doctor and the clinical environment, and the use of very junior doctors as facilitators of this learning.  For two academic years, all first-year medical students at UCL Medical School shadowed a Foundation Year (FY) doctor for a four-hour shift to experience and understand the work of junior doctors. Feedback from students and FY doctors was gathered and analysed. | The students found the FY doctors to be good near-peer tutors and enjoyed exploring the clinical environment, but felt that the unstructured learning environment was difficult. The FY doctors felt that learning in and about the clinical environment was an important learning outcome for the students, although they found supervising junior medical students in a shadowing context difficult. | FY doctors are an effective and underutilised resource in introducing novices to the role of a medical professional in the clinical environment; however students and FY doctors need support to maximise the learning potential of early shadowing activities. | MERSQI: 9  COREQ: 633-637 |
| 77 | Lovell, 2016 | How to build a resilient surgeon: communities, cultures and complexity | Review paper | Not indicated | Not indicated | Not indicated | NA |
| 78 | Berman et al., 2008 | Attracting Surgical Clerks to Surgical Careers: Role  Models, Mentoring, and  Engagement in the  Operating Room | Quantitative | This study was designed to identify those aspects of the clerkship that are associated with medical students expressing an interest in surgery at the end of the clerkship. | Students who sutured (p=001), drove the camera (p =01), stated that they felt involved in the operating room (p =009), and saw residents (p =03) and attendings (p =0003) as positive role models were more likely to be interested in surgery. After adjusting for covariates, students who sutured in the operating room were 4.8 times as likely to be interested in surgery (95% CI, 1.5 to 14.9) and students who drove the camera were 7.2 times as likely to be interested in surgery (95% CI, 1.1 to 46.8). | Students who participate actively in the operating room and those who are exposed to positive role models are more likely to be interested in pursuing a career in surgery. To optimize students’ clerkship experiences and attract top candidates to the field of surgery, clerkship directors should encourage meaningful engagement of students in the operating room and facilitate mentoring experiences. | 12 |
| 79 | Ratanawongsa  et al., 2005 | Third-year medical students' experiences with dying patients during the internal medicine clerkship: a qualitative study of the informal curriculum | Qualitative | To explore third-year medical students’ experiences with death and dying patients during the first internal medicine clerkship. | Twenty-eight (87.5%) students participated in interviews. All students encountered death or dying patients, and most cared directly for at least one dying patient. Students’ relationships with patients were characterized by attachment, empathy, and advocacy. Students valued preparation by preclinical end-of-life (EOL) courses, but assigned greater value to patient care experiences guided by teams that acknowledged deaths, role modeled EOL care, and respected students’ participation in patient care. Clerkship experiences in EOL care affected students’ developing professional identities by affording opportunities to manage strong emotions, understand the challenges of transitioning to residency, and gain a sense of self- efficacy as future physicians providing EOL care. | Third-year medical students’ experiences with dying patients affect their skills and attitudes in EOL care, as well as the emergence of their professional identities. The behaviors and attitudes modeled by residents and attendings during the clerkships can strongly influence students’ perceptions of and self-efficacy in EOL care. Further research and interventions into how residents and attendings model responses to death in the clinical clerkship may suggest strategies not only for EOL training, but also for mentoring professional development. | 641-646 |
| 80 | Nieman et al., 2006 | The Impact of Preclinical  Preceptorships on  Learning the  Fundamentals of Clinical Medicine and Physical  Diagnosis Skills | Quantitative | The study aims to find out if preclinical primary care preceptorships have a positive effect on medical students’ clinical performance.    Students’ performances were assessed using a written examination and clinical assessment (OSCE). “After adjusting for factors that might have explained differences in the students’ performances, logistic regression models were used to assess the association of the outcome variables with participation in a preceptorship.” | Preliminary evidence indicates that a short preclinical primary care preceptorship can help medical students to consolidate and integrate the fundamental cognitive and clinical skills they will apply during the clinical years of medical training. | Preceptorship has a positive effect on students’ cognitive and clinical skills. | NA |
| 81 | Billett S (2002) | Workplace pedagogic practices: Participation and learning | Descriptive | This paper advances conceptual tentative bases for understanding workplace pedagogic practices. It proposes that whether arising through everyday work activities or guided learning in workplaces, learning is shaped by workplace participatory practices. This learning is held to be co-participative: the reciprocal process of how the workplace affords participation and therefore learning, and how individuals elect to engage with the work practice | NA | NA | NA |
| 82 | Golden et al.,  2018 | Learning to be a doctor: Medical students’ perception of their roles in longitudinal outpatient clerkships | Qualitative | To understand how medical students perceive their roles in early longitudinal primary care clerkships.  Medical students enrolled in one of two longitudinal primary care clerkships – Education Centered Medical Home (ECMH) or Individual Preceptorship (IP) – participated in semi-structured interviews. Interviews were recorded, transcribed, and analyzed using a grounded theory and constant comparative approach. | Students (N=35) in both clerkships perceived benefits of early clinical exposure, reflecting positively on having time to interact with patients. Identified roles ranged from shadower to collaborator to diagnostician; a progression from position-centered to more patient- centered roles emerged. ECMH students also identified as health educators, care managers, and mentors. IP students described the clerkship primarily as an opportunity to acquire clinical knowledge and practice skills, expressing perceptions of being a transient “visitor” in the clinic, whereas ECMH students reported taking an active role in continuity care of patients. | Students identified benefits early longitudinal outpatient primary care clerkships, supporting the inclusion of these experiences within medical school curricula. Clerkships with an emphasis on longitudinal and team-based care may further promote student participation in patient care and professional development. | COREQ:  1, 5-18 |
| 83 | Lim-Dunham et al., 2016 | A Vertically Integrated  Online Radiology  Curriculum Developed as a Cognitive  Apprenticeship: Impact on  Student Performance and  Learning | Mixed methods | We describe the development of the vertical online radiology curriculum and evaluate its impact on student achievement and learning process using a mixed method approach. The curriculum was developed over a 2-year period. Student participation was voluntary in the first year and mandatory in the second year. For quantitative curriculum evaluation, student metrics for voluntary versus mandatory groups were assessed using independent sample t tests and variable entry method regression analysis. For qualitative analysis, responses from a survey of students about the value of the curriculum were organized into defined themes using consensus coding. | Mandatory participation significantly improved (p = .001) the mean radiology examination score (82 %) compared to the voluntary group (73%), suggesting that mandatory participation had a beneficial effect on student performance. Potential preexisting differences in underlying general academic performance were accounted for by including mean basic science grades as the first variable in the regression model. The significant increase in R2 from .16 to .28 when number of radiology cases completed was added to the original model, and the greater value of the standardized beta for this variable, suggest that the curriculum made a significant contribution to students’ radiology examination scores beyond their baseline academic  performance. Five dominant themes about curricular characteristics that enhanced student learning and beneficial outcomes emerged from consensus coding. These themes were (1) self-paced design, (2) receiving feedback from faculty, (3) clinical relevance of cases, (4) gaining confidence in interpreting radiological images, and (5) transfer of conceptual knowledge to actual practice. | The vertically integrated online radiology curriculum can positively impact student performance and learning process in the context of the cognitive apprenticeship model. | COREQ: 1-6 MERSQI: 12.5 |
| 84 | Thompson et al., 2016 | A clinical procedures curriculum for undergraduate medical students: the eight-year  history of a third-year immersive experience | Qualitative | The primary educational objective of this program was to formally introduce medical students to clinical procedures thought to be important for success in residency. The immersion strategy (teaching numerous procedures over a 7-day period) was intended to complement the student’s education on third-year core clinical clerkships.  The course introduced 27 skills over 7 days. Teaching and learning methods included lecture, prereading, videos, task trainers, peer teaching, and procedures practice on cadavers. In year 4 of the program, a peer-team teaching model was adopted. We analyzed program evaluation data over time. | Students valued the selection of procedures covered by the course and felt that it helped prepare them for residency (97%). The highest rated activities were the cadaver lab and the advanced cardiac life support (97 and 93% positive endorsement, respectively). Lectures were less well received (73% positive endorsement), but improved over time. The transition to peer-team teaching resulted in improved student ratings of course activities (p<0.001). | A dedicated procedural skills curriculum successfully supplemented the training medical students received  in the clinical setting. Students appreciated hands-on activities and practice. The peer-teaching model improved course evaluations by students, which implies that this was an effective teaching method for adult learners. This course was recently expanded and restructured to place the learning closer to the clinical settings in which skills are applied. | NA |
| 85 | Rangachari et al, 2017 | Clinical coaching: Evolving the apprenticeship model for modern housestaff | Review Paper | Feedback is one of the core components of  teaching in the clinical setting. Traditionally, this activity has emphasized observations made by senior physicians and delivered to medical trainees. However, the optimal approach to feedback remains uncertain, and the literature abounds with trainee-perceived inadequacies in feedback content, quality, and impact. Moreover, given the multiplicity of demands on trainees and their physician mentors, we propose that medical trainees themselves—specifically, medical residents—are poised to serve as unique adjunct effectors of feedback. We propose a model of “clinical coaching” for residents as teachers, with emphasis on the active roles of both the feedback “giver” and “recipient”. By working to better recognize and prepare residents for this vital role, we propose to encourage efforts to optimize the structure, execution, and impact of feedback in the contemporary climate of medical education. | Not indicated | The steps proposed in our model provide a strategy for promoting resident-led clinical coaching as a means to enhance the ongoing assessment and effective feedback that are central to medical education. While teaching, coaching, and mentorship were inseparable activities when residency training first began, the entirety of these responsibilities can no longer be shouldered by attending physicians alone. Like Osler, we have the opportunity to restructure apprenticeship for our era. | NA |
| 86 | Iyasere, C. A., et al. (2016) | Beyond Continuing Medical Education: Clinical Coaching as a Tool for Ongoing Professional Development | Qualitative | For most physicians, the period of official apprenticeship ends with the completion of residency or fellowship, yet the acquisition of expertise requires ongoing opportunities to practice a given skill and obtain structured feedback on one's performance.  In July 2013, the authors developed a clinical coaching pilot program to provide early-career hospitalists with feedback from a senior clinical advisor (SCA) at Massachusetts General Hospital. A Hospital Medicine Unit-wide retreat was held to help design the SCA role and obtain faculty buy-in. Twelve SCAs were recruited from hospitalists with more than five years of experience; each served as a clinical coach to 28 early-career hospitalists during the pilot. | Of 25 responding early-career hospitalists, 23 (92%) rated the SCA role as useful to very useful, 20 (80%) reported interactions with the SCA led to at least one change in their diagnostic approach, and 13 (52%) reported calling fewer subspecialty consults as a result of guidance from the SCA. In response to questions about professional development, 18 (72%) felt more comfortable as an independent physician following their interactions with the SCA, and 19 (76%) thought the interactions improved the quality of care they delivered. | To better understand the impact and generalizability of clinical coaching, a larger, longitudinal study is required to look at patient and provider outcomes in detail. Further refinement of the SCA role to meet faculty needs is needed and could include faculty development. | NA |
| 87 | [Liselotte Dyrbye](https://pubmed.ncbi.nlm.nih.gov/?term=Dyrbye+L&cauthor_id=26695473), [Tait Shanafelt](https://pubmed.ncbi.nlm.nih.gov/?term=Shanafelt+T&cauthor_id=26695473), (2016) | A narrative review on burnout experienced by medical students and residents | Review | This review summarises reporting on burnout among medical students and residents (trainees). MEDLINE was searched for peer-reviewed, English language articles published between 1990 and 2015 reporting on burnout among trainees. The search used combinations of Medical Subject Heading terms medical student, resident, internship and residency, and burnout, professional. | Studies suggest a high prevalence of burnout among trainees, with levels higher than in the general population. Burnout can undermine trainees' professional development, place patients at risk, and contribute to a variety of personal consequences, including suicidal ideation. Factors within the learning and work environment, rather than individual attributes, are the major drivers of burnout. | Multi-pronged efforts, with attention to culture, the learning and work environment and individual behaviours, are needed to promote trainees' wellness and to help those in distress. | NA |
| 88 | Linnet et al.,  2012 | Cognitive Apprenticeship Learning in Paediatric Clinical Settings | Qualitative | Cognitive apprenticeship learning focuses on improving understanding and clinical diagnostic reasoning among learners. The aim of this study was to describe the feasibility of cognitive apprenticeship encounters in clinical paediatric settings in terms of time consumption; and to describe the encounters in terms of learner and teacher behavior.  This study was based on data from 485 self-reports filled in by participating learners during a 3-month quality improvement project conducted at three Danish paediatric departments. Teacher and learner cognitive apprenticeship skills training took place at repeated workshops. A total of 59 different learners participated, 4 were medical students, 49 were residents, and 6 were specialists in pediatrics. A total of 75 different teachers participated. | Apprenticeship encounters between teachers and learners lasted median 9 (range 2-120) minutes. In 47% of the encounters, the teacher and learner examined the child together. In 91% of the encounters, the learner reported that his or her knowledge was challenged and in 92% of the encounters, the learners and the teachers verbalized their clinical diagnostic reasoning processes. Educational strategies to reach learning objectives were reported in 77%. | Cognitive apprenticeship learning was a feasible approach to teaching and learning in paediatric departments. Median 9 (range 2-120) minutes were used for the encounters. Almost all learners reported being challenged during the encounter. Almost half of the encounters included a joint teacher and learner examination of a patient. | NA |
| 89 | Tariq et al. 2020 | Using the cognitive apprenticeship model to identify learning strategies that learners view as effective in ward rounds | Qualitative | Cognitive apprenticeship model (CAM) is an instructional model for situated learning. There is limited data available on application of the CAM in clinical settings. The aim of the study was to identify learning strategies using CAM, which in the opinion of learners are effective in ward rounds. Participants were residents and medical students who rotated through internal medicine at Aga Khan University Hospital, Karachi. We sought learners’ opinion on a structured questionnaire based on four principal dimensions of cognitive apprenticeship. A previously determined set of 10 defined competencies were compared with CAM’s six teaching/learning (T/L) methods (modelling, coaching, scaffolding, articulation, reflection and  exploration) as well. Mean and SD were calculated. Mann-Whitney test was used to compare scores. | Of 195 participants, there were 100 men (51.3%) and 95 women  (48.7%). Perceived learning for six  T/L methods, ranged from 3.7 to 3.9 (max=5). Coaching and scaffolding had the highest scores. Statistically significant difference between the student and resident groups was noted. Medical students perceived coaching and scaffolding (4.1 and 4.05) and residents rated coaching, articulation and exploration as most effective (3.9 and 3.8). Majority (82.1%) reported a positive learning environment in wards. | CAM enabled to identify two T/L methods (scaffolding and coaching) that are important for learning in ward round. Limited differences in perceived effectiveness of the T/L methods indicate that variety can be used to sustain interest in learners. Positive learning environment, team diversity and tasks of increasing complexity contribute to learning. | NA |
| 90 | Liljedahl M et al. (2021) | How workplace learning is put into practice: contrasting the medical and nursing contexts from the perspective of teaching and learning regimes | Qualitative | This study aimed to explore practices of workplace learning in the two adjacent healthcare professions; medicine and nursing. Field observations and follow-up interviews were performed in three clinical departments and the data set comprised 12 full days of observations and 16 formal follow-up interviews. | Four teaching and learning regimes were found in the data. In the medical context, workplace learning was either practiced as reproduction of current practice or through stimulation of professional development. In the nursing context, workplace learning was either based on development of partnership between student and supervisor or on conditional membership in a professional community. | The medical and nursing contexts demonstrated varying underpinnings and assumptions relating to teaching and learning. The respective practices of workplace learning in the medical and nursing context appear to hold substantial differences which might have implications for how we understand practices of workplace learning. | NA |
| 91 | Schmidt et al.,  2020 | Deliberate Practice:  Applying the Expert Performance Approach to  Gynecologic Surgical  Training | Review paper | Surgical training is shifting from the historical Halstedian apprenticeship model to outcomes-based methods. Surgical residents can reach a higher level of performance when utilizing deliberate practice and the expert performance approach. This article discusses methods for implementing deliberate practice and the expert performance approach into gynecologic surgical training programs. | The current surgical training model blends the Halstedian apprenticeship  model with an outcomes-based approach. Trainees still utilize traditional and purposeful practice techniques to acquire new skills. Current evidence shows that deliberate practice and the expert performance approach can be used to better train the next generation of gynecologic surgeons. | A focus on developing domains of surgical practice and integrating critical behaviors of expert surgeons will allow residents to become competent with the potential to reach mastery levels of performance. Training programs should implement the use of structured rating scales, standardized simulation-based skills curricula, and video reviews to enhance resident surgical training. Surgical coaches should be developed and utilized to enable trainees to reach a higher level of performance. | NA |
| 92 | Behling et al.,  2021 | One-on-one mentoring for final year medical students during the neurosurgery rotation | Quantitative | We developed a one-on-one mentoring program to allow individually tailored teaching of clinical practical skills to support final year students with varying skill sets during their neurosurgical clinical clerkship. Each participating student (n = 23) was paired with a mentor. At the beginning students were asked about their expectations, teaching preferences and surgical interest. Regular meetings and evaluations of clinical practical skills were scheduled every 2 weeks together with fixed rotations that could be individually adjusted. The one-on-one meetings and evaluations with the mentor gave each student the chance for individually tailored teaching. After completion of the program each student evaluated their experience. | The mentoring program was well received by participating students and acquisition or improvement of clinical practical skills was achieved by most students. A varying practical skill level and interest in the field of surgery was seen. | A neurosurgical one-on-one mentoring program is well received by final year medical students and allows for individually tailored learning of clinical practical skills. | 6 |
| 93 | [Danielle L Terry](https://pubmed.ncbi.nlm.nih.gov/?term=Terry+DL&cauthor_id=39493933), [Prabhdeep Bajwa](https://pubmed.ncbi.nlm.nih.gov/?term=Bajwa+P&cauthor_id=39493933), (2024) | Associations Between Supervisory Alliance, Medical Resident Distress, Burnout, and Self-Esteem | Quantitative | While it is known that the medical training environment hosts high rates of burnout, little research has examined the role of supervisory alliance in regard to burnout, distress, and self-esteem. This study aimed to apply previous models of supervisory alliance, including those within apprenticeship programs, to medical education, and explore associations between educational alliance and trainee self-esteem, burnout, and distress. Participants included 108 medical trainees (response rate = 81%). Medical trainees were recruited from a rural Pennsylvanian teaching hospital and asked to complete a voluntary and anonymous electronic survey. Multiple linear regression was used to examine the association between supervisory alliance and burnout and distress, while controlling for average hours worked and slept. | Results suggested that supervisory alliance was associated with burnout, distress, and self-esteem among medical trainees. | Given the high rates of depression and burnout among medical providers, it may be helpful to understand the factors that may impact medical trainee's distress and esteem. Future longitudinal research might examine the role of the supervisory alliance over time, and whether strong mentorship and guidance may serve to protect trainees from burnout throughout training and into their career. | NA |
| 94 | Coakley et al. (2023) | Experience of enhanced near-peer support for new medical graduates of an Irish university: a phenomenological study | Qualitative | To explore the experience of commencing practice for these new doctors with this increased near-peer support. Participants recorded audio diaries from their commencement of work, and a semistructured interview was conducted with each, after 3 months, concerning their experience of their overlap with the previous year's interns. | Participants felt reassured by having a near-peer in the same role and safe to seek their support. This empowered them to gradually assume increasing responsibility and to challenge themselves to further their learning. Participants perceived that commencing work before the annual change-over of other grades of doctor-in-training enhanced their professional identities and improved patient safety. | Enhanced near-peer support for new doctors offers a potential solution to the stressful transition to practice. Participants were legitimate members of the community of practice, with the status and responsibility of first-year doctors. Furthermore, this study reinforces the benefit of asynchronous job change-over for doctors-in-training. | NA |
| 95 | Wallenburg et al., 2016 | Repairing reforms and transforming professional practices: A mixed-methods analysis of surgical training reform | Mixed methods | This article investigates the understudied reform of postgraduate medical education. The reform takes in a shift from apprenticeship-based training based on ‘learning-by-doing’ and socialization to time-restricted, streamlined, competency-based training programs based on competency-based training and standardized performance assessment.  In this study, the qualitative data consists of semi-structured in-depth interviews with attending surgeons and surgical residents, and participant observations of monthly meetings of the regional training committee.  A questionnaire distributed among all residents and attending surgeons in the training region where the study was conducted provided the quantitative data. | A majority stated that the traditional training model forms an essential part of surgical residency training (residents 86%, mean ¼ 4.26 versus attendings 78%, mean ¼ 3.89). The statement that the new training program is an improvement compared to the traditional master-apprenticeship model was in general rejected (residents 14%, mean ¼ 2.93 versus attendings 21%, mean ¼ 2.93).  Only 14% of residents (mean ¼  2.26) and 11% of attending surgeons (mean ¼ 2.16) agreed with the statement ‘fellowships will be superfluous in the future’, indicating the belief that when residents qualify they are not ready to act as fully competent surgeons in their field of expertise. | Our mixed-methods study reveals that both surgeons and surgical residents are rather ambivalent towards the reform. On the one hand, they perceive the increasing structuring and formalization of surgical training as a necessary answer to the new regulations (i.e. limitation of duty hours, shortening of the training program) due to which residents spend far less time on the work floor and have less contact with their attendings. The new training methods, like formal assessment procedures, are considered a feasible and even necessary answer to this. Yet, on the other hand the reforms are criticized as they impede on the highly valued tradition of embedded training, socialization and gradual integration in the medical community | MERSQI: 1, 3-15 COREQ:14 |
| 96 | Knfe, G., et al. (2024) | Surgical residents' perceptions of the operating theatre educational environment at St. Paul's Hospital Millennium Medical College: A cross-sectional survey | Quantitative | This study aimed to assess surgical residents' perceptions of the operating theatre educational environment and associated factors in the surgical department at St. Paul's Hospital. This cross-sectional study was conducted in March 2022 among surgical residents at St. Paul's Hospital Millennium Medical College to assess their perceptions of the operating room educational environment using the OREEM questionnaire. | The overall mean score was 69% with subscale scores for teaching and training at 47.9/65.0 (73.7%), learning opportunities at 34.5/55.0 (62.7%), the atmosphere at 28.9/40.00 (72.4%) and workload/supervision/ support at 27.5/40.0 (68.7%). Male and female residents differed significantly in perceptions of “atmosphere” (t_127_ = 3.35, p < 0.001) and in junior versus senior residents' perceptions of the “learning opportunities” and “atmosphere” at p-values of 0.023 and 0.028 respectively. | Residents had positive perceptions of their training and teaching, learning opportunities, the atmosphere in the operation theatre, and the supervision they received in the operation theatre. The operating room's “teaching and training” component received the highest score, while the operating room's “learning opportunities” component received the lowest. This indicates the importance of establishing a positive learning environment with sufficient “hands-on” experience, especially during emergencies. In addition, preoperative planning, case discussions, and feedback after the surgery should be routine. | NA |
| 97 | Adonizio et al.,  2012 | Implementing a longitudinal integrated clerkship (LIC) for the third year at the common wealth medial college | Mixed methods | Explores the outcomes of the curricular model of longitudinal integrated clerkship (LIC). Evaluation encompasses: logging of all clinical encounters into an e-portfolio, OSCEs, NBME content exams, faculty evaluations at 30 days, 6 months and end of year, measures of professionalism including cultural competency and empathy. | Not indicated | This study seeks to shed light on the potential of the LIC. Recent reports from the Macy and Carnegie  Foundation support the expansion of the longitudinal integrated clerkship as a model that supports longitudinal exposure to patients and faculty, enhances patient-centered attitudes and the maintenance of empathy, while demonstrating equivalent or better outcomes on measures such as written  examinations and OSCEs compared  with traditional inpatient block clerkships. Despite this, this curricular model is underused. | COREQ: 1-2 MERSQI: 10.5 |
| 98 | Brody et al.,  2004 | Promoting the  Development of Doctoring  Competencies in Clinical  Settings | Quantitative | This UME-21 project was developed to promote a variety of clinical competencies during a 12week medicine clerkship for third-year students.    The clerkship is divided into three 4-week rotations—two inpatient rotations and one outpatient rotation. During each rotation, students select a competency, review the module about that competency on the clerkship Website, and perform a literature search.    Learning exercises prompt students to ask their preceptor to model and discuss the performance of the competency on at least one patient and to provide feedback on their performance at least twice.    At the end of each rotation, students are required to write about what they learned from the articles they read, write a critical analysis of their performance of the competency on one patient, and complete an evaluation  questionnaire. This report is based on the results from the students’ evaluation questionnaire. | At the end of the first six rotations, 120 students completed 330 evaluations of the course (93% response rate).    The most frequently selected competency modules were behavior modification and patient education.    In 81.5% of the evaluations, students felt that there was at least moderate improvement in their ability to perform the selected competency during the rotation.    By the end of the rotation, in 85.3% of the evaluations, students indicated that they were confident performing the competency most or almost all of the time.    Observing the preceptor was the component of the curriculum most often rated as helpful (59.1%), followed by literature review(57.9%), reviewing the Website module (45.2%), and observation and feedback by the preceptor (32.7%) | Based on student reports, the approach described in this paper appears to be a promising way to teach important doctoring competencies in a clinical setting. | MERSQI: 12 |
| 99 | Eltayar, A. N., et al. (2020) | Online faculty development using cognitive apprenticeship in response to COVID-19 | Review | Despite the coronavirus disease 2019 (COVID‐19) pandemic, it is still necessary to develop good assessment tools to evaluate the competence of our students. Our medical education department, therefore, changed its face to face workshops on 'how to create multiple choice questions (MCQs)' into online ones. It was the first time our medical education department had created questions online. | Cognitive apprenticeship concepts were adopted when designing and implementing the training workshop. We started with full instructions on how to use Zoom™ cloud (Zoom Video Communications Inc., San Jose, CA, USA) through video and a technical training session offered before the workshop. Video‐recording of a lecture explained the guidelines adopted by the assessment unit regarding the MCQ design. A document including frequently asked questions regarding the design of MCQs was also disseminated 2 days before the synchronous meeting. | Proper design of hands‐on activities can change faculty staff minds.  Offering multiple and different opportunities for hands‐on activities to the trainee with constructive feedback can improve the outcomes of faculty development activities.  It is important to provide appropriate scaffolding to faculty staff when they perform assigned activities to support their learning.  Providing additional experience to practice (and check ability to transfer newly acquired knowledge and skills) is valuable.  Unexpectedly, when we were able to achieve what had been done in previous face to face training sessions, trainees reported less interference and better participation was achieved by conducting the workshop online. | NA |
| 100 | Thiedke et al.,  2004 | Student observations and ratings of preceptor's interactions with patients: the hidden curriculum | Mixed methods | Professional values and behavior are conveyed to students through both formal and informal curricular activities. This study examined 1styear students’ observations of community-based physicians’ behaviors during a community-based clinical experience. | 119 students completed the instrument (87% response rate). Students rated aspects of physicians’ demeanor with patients highest (mean ranges 4.7–4.57). The lowest rated item was the physician’s view of his or her professional role (M = 3.39), eliciting patients’ ideas about illness and treatment (M = 3.55), and modeling inter- viewing techniques learned in class (M = 3.71). | Community-based physicians reinforce many professional values associated with positive role-modeling aspects of the physician–patient interaction. | MERSQI: 11 COREQ: NA |
| 101 | Côté et al.,  2014 | Preceptors’ Understanding and Use of Role Modeling to Develop the CanMEDS  Competencies in  Residents | Qualitative | The purpose of this study was to describe how preceptors understand and use role modeling to develop CanMEDS competencies in residents. In 2010, the authors conducted a descriptive qualitative study with preceptors in medical, surgical, and laboratory specialties who supervised residents on a regular basis at the Université Laval Faculty of Medicine (Québec,  Canada). Respondents participated in semistructured, individual interviews. An inductive thematic analysis of interview transcripts was conducted using triangulation. | Most participants highlighted the importance of role modeling to support residents' development of the CanMEDS competencies, particularly communication, collaboration, and professionalism, which preceptors perceived as "less scientific" and the most difficult to teach. Although most participants reported using an implicit, unstructured role modeling process, some described more explicit strategies. Eight types of educational  challenges in role modeling the CanMEDS competencies were  identified, including encouraging reflective practice, understanding the competencies and their importance in one's specialty, and being aware of one's strengths and weaknesses as a clinical teacher. | Preceptors are aware of the importance of role modeling competencies for residents, but many do so only implicitly. This study's findings are important for improving strategies for role modeling and for the professional development of preceptors. | 934-938 |
| 102 | Shah et al.  2020 | Role modelling and active  apprenticeship during the  COVID-19 pandemic | Reflection | Not indicated | Not indicated | Trainees can develop skills like effective communication, empathy and shared decision making from observing attitudes of role models during this difficult hour of the pandemic trainees will remember how the commitment and the compassion of their role models reinfused them with enthusiasm to give their best when it was needed the most. | NA |
| 103 | Stalmeijer et al.  2013 | Clinical Teaching Based on Principles of Cognitive Apprenticeship: Views of Experienced Clinical Teachers | Qualitative | To explore (1) whether an instructional model based on principles of cognitive apprenticeship fits with the practice of experienced clinical teachers and (2) which factors influence clinical teaching during clerkships from an environmental, teacher, and student level as perceived by the clinical teachers themselves. The model was designed to apply directly to teaching behaviors of clinical teachers and consists of three phases, advocating teaching behaviors such as modeling, creating a safe learning environment, coaching, knowledge articulation, and exploration.  A purposive sample of 17 experienced clinical teachers from five different disciplines and four different teaching hospitals took part in semi-structured individual interviews. Two researchers independently performed a thematic analysis of the interview transcripts. Coding was discussed within the research team until consensus was reached. | All participants recognized the theoretical model as a structured picture of the practice of teaching activities during both regular and senior clerkships. According to participants, modeling and creating a safe learning environment were fundamental to the learning process of both regular and senior clerkship students. Division of teaching responsibilities, longer rotations, and proactive behavior of teachers and students ensured that teachers were able to apply all steps in the model. | The theoretical model can offer valuable guidance in structuring clinical teaching activities and offers suggestions for the design of effective clerkships. | NA |
| 104 | Régo et al.,  2009 | Using a structured clinical coaching program to improve clinical skills training and assessment, as well as teachers' and students' satisfaction | Quantitative | The ability to deliver the traditional  apprenticeship method of teaching clinical skills is becoming increasingly more difficult as a result of greater demands in health care delivery, increasing student numbers and changing medical curricula. Serious consequences globally include: students not covering all elements of clinical skills curricula; insufficient opportunity to practise clinical skills; and increasing reports of graduates’ incompetence in some clinical skills.  A systematic Structured Clinical Coaching Program (SCCP) for a large cohort of Year 1 students was developed, providing explicit learning objectives for both students and paid generalist clinical tutors. It incorporated ongoing multi-source formative assessment and was evaluated using a case-study methodology, a control-group design, and comparison of formative assessment scores with summative Objective Structured Clinical Examination (OSCE) scores. | Students demonstrated a higher level of competence and confidence, and the formative assessment scores correlated with the Research students’ summative OSCE scores. SCCP tutors reported greater satisfaction and confidence through knowing what they were meant to teach. At-risk students were identified early and remediated. | The SCCP ensures consistent quality in the teaching and assessment of all relevant clinical skills of all students, despite large numbers. It improves student and teacher confidence and satisfaction, ensures clinical skills competence, and could replace costly OSCEs. | 12.5 |
| 105 | Sen Gupta et al.,  2008 | Rural internships for final year students: clinical experience, education and workforce | Mixed methods | The James Cook University School of Medicine is the only complete medical school in northern Australia, and it has a mission to prepare graduates to meet the unique needs of the region with a particular emphasis on rural, remote, Indigenous and tropical health. This article describes the development, implementation and assessment of the first years of the program, from 2005 to 2007. Evaluation included student questionnaires, site visits and interviews, and follow-up teleconferences with preceptors. | The rural internship provides senior medical students with valuable experience by active participation in the healthcare team. Students reported a rich and varied clinical experience. Students accept limited supervised responsibility and further their ability and confidence to undertake the role of the intern. Importantly, they proved not to be a burden to the system. This rotation therefore appears to meet educational needs without compromising the local workforce (and indeed may add to it). Students felt welcomed by their communities and enjoyed the social and cultural aspects of their attachment, as well as the clinical aspects and the opportunity to further their understanding of rural communities, rural health care and the healthcare team. Preparation of the students, the preceptors and the communities emerged as a key element of success | This model extends and enhances the traditional apprenticeship model by its rural focus and distributed nature, and involvement of the entire student cohort. In addition, the contribution to patient care by senior students and junior doctors enables a consultant-registrar-resident model, in which experienced rural doctors function as consultants providing advice, support and tuition rather than predominantly face-to-face patient care. This approach also provides a means to address an emerging paradox: rural preceptors and communities want to teach students, appreciating the longterm workforce implications, but are increasingly constrained by resources, particularly time. Similar innovative approaches should be explored in other settings. | MERSQI: 1-8 COREQ:9.5 |
| 106 | Newcomb et al. 2018 | Evaluating Surgical  Residents' Patient-  Centered Communication  Skills: Practical  Alternatives to the  "Apprenticeship Model" | Qualitative | The Accreditation Council for Graduate Medical Education (ACGME) requires residency programs to assess communication skills and provide feedback to residents. We aimed to develop a feasible data collection process that generates objective clinical performance information to guide training activities, inform ACGME mile- stone evaluations, and validate assessment instruments. Residents care for patients in the surgical clinic and in the hospital, and participate in a communication curriculum providing practice with standardized patients (SPs). We measured perception of resident communication using the 14-item Communication Assessment Tool (CAT), collecting data from patients at the surgery clinic and surgical wards in the hospital, and from SP encounters during simulated training scenarios. We developed a hand- out of CAT example behaviors to guide patients completing the communication assessment. | The primary outcome is the percentage of total items patients rated “excellent;” we collected data on 24 of 25 residents. Outpatient evaluations resulted in significantly higher scores (mean 84.5% vs. 68.6%, p o 0.001), and female patients provided nearly statistically significantly higher ratings (mean 85.2% vs. 76.7%, p 1⁄4 0.084). In multivariate analysis, after controlling for patient gender, visit reason, and race, (1) residents’ CAT scores from SPs in simulation were independently associated with communication assessments in their concurrent patient population (p 1⁄4 0.017), and (2) receiving CAT example instructions was associated with a lower percentage of excellent ratings by 9.3% (p 1⁄4 0.047). | Our data collection process provides a model for obtaining meaningful information about resident communication proficiency. CAT evaluations of surgical residents by the inpatient population had not previously been described in the literature; our results provide important insight into relationships between the evaluations provided by inpatients, clinic patients, and SPs in simulation. Our example behaviors guide shows promise for addressing a common concern, minimizing ceiling effects when measuring physician-patient communication. | NA |
| 107 | DeVita, 2007 | Simulation and the Prognosis for the Apprenticeship Model of Health Care Education | Editorial | The article discusses the current model of apprenticeship and the promise of simulation education | Not indicated | Mainstreaming simulation is necessary. Of course, we should recognize that advances in simulation technology will be needed to encompass a more complete set of skills and experiences so as to enable the goals of more efficient, effective, and safe education. | NA |
| 108 | Chiu et al. (2015) | Experience of moral distress among pediatric surgery trainees | Quantitative | The aim of this study was to identify and qualify the ethical dilemmas faced by paediatric surgery trainees in an apprenticeship program. An online survey was sent to paediatric surgery trainees graduating between 2005 and 2008. Consent was obtained, and study investigators were blinded to the identity of the respondents during data analysis. | Of the 40 respondents, only 59% felt they had received adequate training in bioethics to handle ethical issues pertaining to the care of critically ill children. Although 83% of respondents routinely participated in palliative care discussions, 30% of respondents desired to have more opportunities to discuss end-of-life issues with their staff. Moral conflicts were resolved through direct discussions with the medical staff, family, or friends. Despite the presence and awareness of institutional policies on ethical behavior, 58% of respondents did not believe that ethical conflicts were resolved as a result of these policies, whereas 31% of respondents felt that reporting of unethical conduct would result in personal reprisals. | Paediatric surgery trainees face ethical and moral conflicts, but some are fearful of reprisals if these concerns are reported. A neutral forum to raise such issues may facilitate open discussions and eventual resolution of these conflicts. | NA |
| 109 | Lyle et al. (2025) | Decreasing Perceived Moral Distress in Pediatrics Residents: A Pilot Study |  | Few studies have identified meaningful methods in reducing moral distress in pediatric trainees. The authors aimed to determine how residents perceive ethics case discussions, whether such a program affects trainee ethics knowledge and perceived moral distress, and if residents' perceived moral distress changes before, during, and after a discussion series. Five separate 1-hour sessions were presented over a 5-month period. Each session consisted of a case presentation by a resident developed under the guidance of an ethicist. | When comparing baseline and final responses, the only significance was increased preparedness to navigate ethical decisions (*p* = 0.004). A 10.2% decline was observed in perceived moral distress. An increase in ethics knowledge was observed. Residents favored case-based, multidisciplinary discussions. Residents desire more sessions, time for small-group discussions, and legal insight. Satisfaction was high with 90.7% of respondents feeling better prepared to address ethical concerns. | Pediatric trainees desire case-based ethics training that incorporates small-group discussions and insight from multidisciplinary topic experts. | NA |
| 110 | [Thomasine K. Kushner](https://www.cambridge.org/core/search?filters%5BauthorTerms%5D=Thomasine%20K.%20Kushner&eventCode=SE-AU) and  [David C. Thomasma](https://www.cambridge.org/core/search?filters%5BauthorTerms%5D=David%20C.%20Thomasma&eventCode=SE-AU)  (2001) | [Ward Ethics](https://www.cambridge.org/core/books/ward-ethics/8C8BA88DCF27710C67BA2E5E24F29573)  *Dilemmas for Medical Students and Doctors in Training* | Review | The most looming obstacle for trainees trying to act morally is the perception of medical hierarchy and struggling to find their place in that hierarchy. Challenging the decisions and actions of someone higher in authority can be the most threatening and unsettling of dilemmas. | NA | NA | NA |
| 111 | [Karen Vaughan](https://www.tandfonline.com/author/Vaughan%2C+Karen), (2017) | The role of apprenticeship in the cultivation of soft skills and dispositions | Qualitative | This article highlighted findings from interviews with GP registrars. They remarked how they needed to apply an ethic of care to themselves so that they could be fully available to their patients. The ethical disposition to engage in continuity of care extended into GPs’ own lives. The were beginning to see that their ability to care deeply for patients depended on taking responsibility for their own wellbeing and avoiding burnout, substance abuse, and apathy or “compassion fatigue”. | NA | NA | NA |
| 112 | Valavanis et al. (2025) | What Is Working in Medical Education: A Call to Action | Review | For learners to be effective and high-quality clinicians, they must learn via apprenticeship, collaborative learning, and use patient centered approach models which may include and identify social determinants of health, trauma informed care, and adverse childhood experiences. The passion must be ignited throughout the learning environment and multiple tools and approaches be utilized so learners are equipped for patients with all forms of challenges. | NA | NA | NA |
| 113 | [Sathyaraj Venkatesan](https://muse.jhu.edu/search?action=search&query=author:Sathyaraj%20Venkatesan:and&min=1&max=10&t=query_term), [Anu Mary Peter](https://muse.jhu.edu/search?action=search&query=author:Anu%20Mary%20Peter:and&min=1&max=10&t=query_term), (2017) | "No time to rest, vent or mourn": Medical Intern Narratives and Graphic Medicine | Review | In spite of major reforms in medical schooling, many significant surveys as well as self-reflective accounts of medical trainees underscore the ongoing prevalence of trainees’ victimization. While expecting an objective approach, ultra-precise logic, and unerring work ethic, the extant medical system often fails to acknowledge the impedances, stress, and unhealthy transformations of interns in the process of becoming a doctor. This essay maps the emotional, psychosomatic, and moral geographies of medical interns/ trainees against the backdrop of bureaucratized centers of professional care. | NA | NA | NA |
| 114 | [R. Abraham](https://www.semanticscholar.org/author/R.-Abraham/26905321), (2015) | Erosion of empathy in medical trainees | Secondary Analysis | Once a student enters the apprentice phase, those scores start to trend downward and continue to decrease during residency. Many hypotheses exist on why this attrition occurs and thereby provide a good starting point to create interventions. | NA | NA | NA |
| 115 | Salas, R. M. E.  et al. (2019) | The PreDoc Program: Pipeline Healthcare Apprenticeship Program through the Lens of a Neurologist | Mixed methods | The PreDoc program is a longitudinal apprenticeship aimed at increasing college student interest in pursuing a healthcare career. This program offers the continuity of clinical, research, and educational exposure in academic medicine utilizing a career immersion approach that allows a graduated level of responsibility, experience, and leadership opportunities. Students get an asynchronous/synchronous curriculum under the direction of academic physicians committed to boosting the pipeline. | Since the implementation of the PreDoc program in 2013, 28 students have enrolled in the program. Twenty-three students completed the survey; 100% ranked the program quality as good/excellent. Students reported more interest in academic medicine (n=19, 83%), neurology (n=18, 78%), and sleep medicine (n=18, 78%). A majority of the students reported that they were extremely likely to pursue a medical career (n=20, 87%). | The PreDoc program has been successful in promoting college student scholarly productivity in healthcare and in garnering student interest in academic medicine, particularly in neurology. | NA |
| 116 | Al-Dubai, (2013) | Emotional burnout, perceived sources of job stress, professional fulfilment, and engagement among medical residents in Malaysia | Qualitative | This study was the first to explore factors associated with emotional burnout (EB) among medical residents in Malaysia. A cross-sectional study was conducted in a universal sample of 205 medical residents in a Malaysian general hospital. The self-administered questionnaire used consisted of questions on sociodemographic and work characteristics, sources of job stress, professional fulfilment, engagement, and EB. EB was measured using the emotional exhaustion subscale, the Maslach Burnout Inventory (MBI). Mean (±SD) age of the respondents was 26.5 (±1.6). | The most common source of job stress was "fear of making mistakes." Most of the participants were dissatisfied with the increase of residentship period from one year to two years. A high level of EB was reported by 36.6% of the respondents. In multivariate analysis, the most important correlates of EB were sources of job stress, professional fulfilment, and engagement. A high prevalence of EB was found among medical residents. Sociodemographic characteristics, performance pressure, and satisfaction with policies were significantly associated with EB. | This study suggests to revert medical residency period to one year. This would enable residency training during undergraduate medical education rather than to impose it after graduation. The role of supervisors should be changed from “evaluators of medical resident competencies” to “mentors” that coach, advice, care, and counsel their medical residents through excellent supervisor-resident rapport. | NA |
| 117 | Harris, 2004 | Professionalism: Part II - Teaching and assessing  the learner's professionalism | Descriptive | Part I of this two-part article introduced the characteristics of professionalism and described how the office-based teacher can be an effective role model. Part II discusses how the preceptor can teach concepts of professionalism during office-based care and assess the learner’s professionalism. | Not indicated | Professionalism is demonstrated and then emulated. Before we can adequately evaluate it, we need to ensure as teachers that we are practicing and reinforcing it. We must also make an effort to observe learners, teach them how to  demonstrate professionalism in specific interactions with patients, and accurately assess their professional behavior. | NA |
| 118 | Health Education England. 2023 | Medical Doctor Degree Apprenticeship: Health Education England 2023 | Web Page | The Medical Doctor Degree Apprenticeship (MDDA) was developed by NHS England with key partners to explore new pathways into medicine which also addressed local workforce challenges. It aimed to provide NHS organisations an innovative way to grow their future medical workforce by recruiting from diverse talent pools within local communities. | NA | NA | NA |
| 119 | [Jason M Satterfield](https://asmepublications.onlinelibrary.wiley.com/authored-by/Satterfield/Jason+M), [Caroline Becerra](https://asmepublications.onlinelibrary.wiley.com/authored-by/Becerra/Caroline), (2010) | Developmental challenges, stressors and coping strategies in medical residents: a qualitative analysis of support groups | Mixed methods | The identification of the longitudinal emotional and developmental coping needs of internal medicine residents could assist in the better design and implementation of supportive interventions. Six internal medicine resident support groups (*n* = 62; residents in postgraduate years [PGY] 1–3) were followed for a period of 2 years. Qualitative data were extracted from monthly support group process notes to identify common themes, stressors, emotions, coping strategies and developmental challenges faced during training. Quantitative questionnaire data were collected on burnout, group attendance and resident satisfaction. | The most common themes included understanding resident roles and responsibilities, developing an identity as a resident and doctor, building professional confidence, cognitive and behavioural responses to stress, and concerns about flaws in local and national health care training and delivery systems. Anxiety and guilt were the most commonly reported emotions, followed by positive emotions and anger.. | Developmentally informed programmatic adaptations could better support the emotional growth and personal and professional development of postgraduate medical trainees. Future directions should include a controlled trial of resident support groups, assessments of ‘active ingredients’ (i.e. to establish which supportive interventions are most effective), and evaluations of programmatic adaptations. | NA |
| 120 | Simpkin et al. 2019 | Identifying central tenets needed in our education systems: Results from a pilot integrated clinical apprenticeship | Qualitative | The ability of healthcare systems to deliver world-class compassionate care depends on the quality of training and education of staff.  Matching student-centered learning with patient-centered care is the focus for much curricula reform. This study explores the effect a novel longitudinal curriculum had on medical students’ attitudes and experiences to better identify central tenets needed in our education system. Single-center, qualitative focus-group study conducted in 2017 of medical students in a longitudinally integrated clinical apprenticeship at a large UK medical school. Students were randomly assigned to focus groups to describe their educational journey and explore how longitudinal learning prepared them for a medical career, valuing their unique position as student participants in the healthcare system. | Results: Four themes emerged from students’ experiences: navigating the patient journey, their professional development, their learning journey, and the healthcare system. | Listening to student voices lends insights for educators refining educational models to produce doctors of tomorrow. This project identified the educational value of students having authentic roles in helping patients navigate the healthcare system and the benefits of consistent mentorship and greater autonomy. The gulf between gaining skills as a future doctor and gaining skills to pass summative exams calls into question assessment methods. | NA |
| 121 | Tsukube et al.,  2020 | The Impact of Cognitive Apprenticeship on the Perceived Growth of Junior Doctors | Quantitative | Although cognitive apprenticeship has been widely used in various educational fields, few empirical studies have examined its  effectiveness in a workplace context. This study aims to investigate the effects of cognitive apprenticeship on junior doctors’ perceived professional growth in hospitals. | The results of multiple regression analyzes showed that all dimensions of cognitive apprenticeship (modeling, coaching, scaffolding,  articulation, reflection and exploration) had positive effects on each doctor’s perceived growth, regardless of the type of clinical practice (surgeon vs physician) or the period of supervision. It was also found that physicians experienced significantly more coaching and scaffolding, and opportunities for, articulation and reflection, than surgeons. | This study confirmed that the six dimensions of cognitive apprenticeship had positive influences on the perceived growth of junior doctors, regardless of their clinical domains or period of supervision and that clinical domains influence the implementation of cognitive apprenticeship. | 10.5 |
| 122 | Rhine et al.,  2021 | Significance of relationships in the cognitive apprenticeship of medical specialty training | Qualitative | To explore how former students of a medical specialty discipline conceived the nature of racial and sociocultural diversity in their learning environment and if/how this influenced their relationships with peers and consultants.  A qualitative enquiry was conducted with 9 formal postgraduate students (registrars) from 6 universities in SA. Data collection was through in-depth individual interviews with open, semi-structured questions. Data were analysed, recognising sub-themes and themes, and interpretation was done in a social  constructionist approach of epistemology, where the participants and researcher co-construct the concepts. | Participants conceived the sociocultural diversity as personal differences and related their experience of not receiving one-on-one mediation or mentoring to a lack of relationship with the consultants,  which was believed to be underpinned by sociocultural differences. Power-plays in departmental culture also inhibited the legitimate access and participation of postgraduate registrars in the community of practice, inhibiting their growth of professional expertise. | Cognitive apprenticeship in medical specialty training has specific challenges in the context where postgraduate students and consultants are from societies previously divided by  inequalities. Common  acknowledgement was that learning in collaboration begins with learning to know each other and by forming relationships. Students faced challenges seeking professional mentorship, which was conceived as a principal contributing factor in their failure to learn. | NA |
| 123 | Wimmers et al., 2006 | Influence of clerkship  experiences on clinical competence | Mixed methods | This study aimed firstly to determine the variation in students’ clinical experiences within and across sites; secondly, to identify the causes of this variation, and thirdly, to investigate the consequences of this variation on students’ competence.  Clerkship students at 12 hospital sites recorded their patient encounters in logbooks. Site characteristics that might influence the variation in patient encounters were collected. Student competence was determined by 3 independent  indicators: a practical end-of-clerkship examination; a theoretical end-of-clerkship examination, and an evaluation of professional performance. A model was developed to test the available clerkship data using structural equation modelling (SEM) software. | Analysis of the logbooks revealed a large variation in the number of patients encountered by students. The average length of patient stay, number of patients admitted, and quality of supervision accounted partly for this variation. An increased number of patient encounters did not directly lead to improved competence. Quality of supervision turned out to be crucially important because it directly impacted student learning and also positively influenced the number of patient encounters. | Monitoring the effectiveness of clerkship by merely asking students to keep a tally of the problems and diseases they encounter, without paying attention to the quality of supervision, does not contribute towards improving student learning. | MERSQI: 11.5  COREQ: 450, 452-453 |
| 124 | Baerheim et al., 2003 | Medical students' evaluation of  preceptorship in general practice in Vestlandet | Qualitative | Placement in general practice is becoming increasingly popular worldwide, and Norwegian study plans include four to eight weeks of such teaching. In this article, we focus on how students experience placement in general practice at the medical school in Bergen, based on a written evaluation form.    In the spring of 2002, 60 last-year medical students in Bergen were placed for four weeks with general practitioners in Western Norway. All students submitted a completed evaluation form for the period. They gave free text answers on how satisfied they were with the plan they received and the guidance they received. | Most students found general medical work surprisingly exciting and educational. The internship teachers were described as very competent, and the work situation was safe and inclusive. A good number of students were observed in clinical work, and received structured feedback from the practice teacher. Many were allowed to influence the work schedule and progression in their own learning. | Although placement in general practice was perceived as instructive and met with great enthusiasm among the vast majority of students, quality assurance work remains to ensure sufficient competence among all practice teachers. | COREQ: 2271-2273 |
| 125 | Goldstein et al., 2005 | Promoting Fundamental  Clinical Skills: A  Competency-Based  College Approach at the University of Washington | Descriptive | The University of Washington School of Medicine has created an integrated developmental curriculum that emphasizes bedside teaching and role modeling, focuses on enhancing fundamental clinical skills and professionalism, and implements these goals via a new administrative structure.  Each medical student is assigned a faculty mentor within a college for the duration of his or her medical school career. Mentors continuously teach and reflect with students on clinical skills development and professionalism and, during the second year, work intensively with them at the bedside.  Competency domains and benchmarks define skill areas in which deepening, progressive attention is focused throughout medical school. | Not indicated | Evaluative data currently being gathered will provide future information concerning the effects of the College system on students’ performance, satisfaction with their education, and the evolution of the third and fourth-year curricula.  We believe, however, that a successful system will consider the need to focus on fundamental clinical skills in a continuous manner across all four years of medical school, to incorporate effective and structured bedside teaching, to use a competency-based model, to commit time and money to an expert group of clinical teachers, and to develop a sustained, dynamic structure of learning that students can internalize for use throughout their careers. | NA |
| 126 | Dornan, 2005 | Osler, Flexner, apprenticeship and ‘the new medical education’ | Article | These are bewildering times for doctors who teach medical students. The UK General Medical Council (GMC) has reminded them they have a duty to teach, yet stirred up such radical change that their task is unrecognizably different from what they themselves experienced as students. This essay draws on biographies of two people who changed the face of medical education in the 20th century to trace the origins of this bewilderment and suggest a direction for the 21st century. Most of today’s doctors just associate the name of William Osler with nodes they were taught about but never see. In Michael Bliss’s biography, he comes to life as a master clinician, apprentice-master, and humanist role model. I first became aware of Abraham Flexner as the author of a century-old report that got medical education into the mess it is in today. Wrong. He was a visionary educationalist who raised standards of medicine round the world by wedding it with biomedical science. After vignettes of these two men, I describe what the GMC has recommended and read between the lines of its recommendations. To finish, I suggest the wheel has come full circle. Apprenticeship, central to Osler and Flexner’s educational visions, needs to be revitalized. | Not indicated | Apprenticeship demands mutual trust and support between teachers and learners, good use of the rich case-mix going through wards, outpatient clinics and health centres, and imaginative capitalization on new collegial structures such as multi-professional teams. The goals of education are too important to be left to chance or whim, so they must be defined rather than left to happenstance, but tacit knowledge and attitudes must be given due recognition and allowed to pass from teacher to learner by role modelling. | NA |
| 127 | Hee, J. M., et al. (2019) | Understanding the Mentoring Environment Through Thematic Analysis of the Learning Environment in Medical Education: a Systematic Review | Qualitative | This systematic review scrutinises reports on learning environments (LE) to extrapolate the findings to the mentoring environment (ME) context to provide a better understanding of ME and their role in the mentoring process. | A total of 4574 abstracts were identified, 90 articles were reviewed, and 58 full-text articles were thematically analysed. The two themes identified were LE structure and LE culture. LE structure regards the framework that guides interactions within the LE. LE culture concerns the values and practices influencing learner-tutor-host organisation interactions. | LE is the product of culture and structure that influence and are influenced by the tutor-learner-host organisation relationship. LE structure guides the evolving tutor-learner-host organisation relationship whilst the LE culture nurtures it and oversees the LE structure. Similarities between LEs and MEs allow LE data to inform programme designers of ME's role in mentoring's success. | NA |
| 128 | Vivekananda-Schmidt et al.,  2011 | Student doctors taking responsibility | Quantitative | New guidelines require all undergraduate medical students to undertake at least one period of assistantship where they assume most of the responsibilities of a first-year graduate doctor (FY1 doctor in the UK) under supervision. To investigate the feasibility of these assistantships. | Competencies that students already engage in as part of existing clinical placements and a number of ‘tacit’ competencies (e.g. practice and promote infection control) were regarded by most as suitable.  Activities that present a clear clinical risk (e.g. prescribing and writing clinical correspondence) were regarded by most as unsuitable or requiring continuous supervision. Some lower risk but hard to measure activities (e.g. responding in practice to audit) were also regarded as unsuitable by some. | The article presents a number of practical issues in relation to assigning responsibility to student doctors. Respondents indicate that successful assistantships will only be possible if the UK National Health Service trusts review their attitude to balancing short- and long-term risks:  assistantships need to be long enough to create genuine responsibility opportunities, and will require investment in supervision beyond the current capacity. | 9.5 |
| 129 | Rassie et al.  2017 | The apprenticeship model of clinical medical education: time for structural change | Reflective | The apprenticeship model, which forms the backbone of the current medical education system, has a strong historical precedent (and indeed multiple strengths). It is, however, important to acknowledge that its application to modern medicine is far from perfect, particularly with the breadth and complexity of current hospital systems. Demands on clinician resources, the sheer volume of knowledge our trainees must amass, short attachments and rigorous assessment schedules are all major challenges to a relatively simplistic educational system. Identifying and addressing these vulnerabilities is essential to enhancing the educational experiences of both undergraduate medical students and junior doctors. The gold standard of good medical education is where students learn the underlying theory and science of a problem at the same time as they encounter that problem in real life”.  The apprenticeship model also allows the student to become familiar with the culture, processes and expectations of the medical workforce. It enables them to become comfortable interacting with patients, and to model their own clinical behaviour on that of respected seniors. | NA | Medical apprenticeship follows a time-honoured tradition, dating back to the Hippocratic Oath era. However, its application to modern medicine is imperfect: thinly-resourced health systems create conflicts between service delivery and dedicated teaching time, short clinical attachments threaten quality longitudinal relationships between teacher and learner, and the value of mentorship is often over- looked. There is a need for structural change, which places increased emphasis on ‘teaching the teacher’, which formally recognises the importance of dedicated teaching and incentivises it accordingly, and which provides structured opportunities for clinical mentor relationships.  Addressing key vulnerabilities in the prevailing apprentice model, and adjusting this model to keep pace with the changing face of healthcare delivery, is essential for the future of medical education. | NA |
| 130 | Caldwell. 2011 | Whatever happened to apprenticeship learning? | Descriptive | Background: I have been a clinical tutor for 10 years in Worthing Hospital, UK. During this time I have seen an increased emphasis on classroom teaching, assessments in controlled situations and simulation, rather than on apprenticeship learning during well-supervised clinical working. Context: At the educational conference on ‘Learning without Leaving the Workplace’ hosted by my hospital, I had an opportunity to present my reflections on apprenticeship or situated learning. This article is a summary of that talk. | Not indicated | The relatively new model of ‘situated learning’ offers an opportunity for academics and clinicians to revitalise the apprenticeship model of learning in, and being stimulated by, the clinical workplace. | NA |
| 131 | Bluemel. 2022 | Doctors need time and support to teach medical students, or no one will learn | Reflective | medical students in the UK feel unprepared for entering practice,2 resulting in many medical schools moving towards an “apprenticeship” or “shadowing” model for the final few months of medical school. This involves integrating students into ward teams by having them shadow foundation year 1 (FY1) doctors, with the aim of equipping the most senior medical students with knowledge of the “ward craft” required for success as a new graduate. These foundation doctors are, however, rarely provided with the support needed to balance teaching work with their clinical roles, as they themselves navigate their first year as a qualified practitioner. With the clinical pressures placed on primary and secondary care at an all time high,3  ward based doctors such as FY1s have increasingly onerous workloads, which push teaching and supervising down their list of priorities. | NA | Medical schools could also consider providing training to foundation doctors who supervise students—especially those involved in “apprenticeship” shadowing. This would allow us to learn about teaching but also gain formal experience to contribute to our foundation portfolios. | NA |
| 132 | Khamishon, R. E., et al. (2025) | Curriculum Innovation: The Osler Apprenticeship in Neurology | Descriptive | Few formal programs provide structured training in medical education (MedEd) early in a learner's career. MedEd training curricula for medical students may cultivate student understanding (and thus interest) in a MedEd career. The Johns Hopkins Osler Apprenticeship (OA) in Neurology is a structured 1-year program for senior medical students (Osler Apprentices/OAs) created to develop talent in MedEd. | Twenty-four of 33 OAs (73%), 18 of 29 alumni (62%), and 6 of 7 preceptors (86%) responded to the surveys. Twenty-one OAs (88%) and 18 alumni (100%) recommended the OA to medical students (level 1). Alumni felt confident participating in educational activities (n = 15/83%), research (n = 15/83%), and leadership (n = 12/67%). Thirty-one OAs (94%) produced educational scholarship (level 2). OAs (n = 22/92%) and alumni (n = 14/78%) agreed that the OA influenced their academic practices and behaviors, and 7 alumni (39%) were recognized or awarded (level 3). Ten alumni (56%) have an educational role (level 4). Preceptors had scholarly output from the OA and reported career or personal development as key factors for participation. | The OA made effective progress toward building a pathway for medical educators while supporting the careers of faculty, allowing for program sustainability. These findings suggest the OA may serve as a prototype for more formalized training programs in neurology MedEd. | NA |
| 133 | Salajegheh, 2023 | Application of cognitive apprenticeship model in clinical education: A scoping review | Review paper | One of the main aspects of medical education is clinical education. Cognitive apprenticeship model as a principle of clinical education provides an opportunity for medical students to transform theoretical knowledge into a variety of cognitive, attitudinal, and psychomotor skills that are essential for patient care. The purpose of this study is to introduce the cognitive apprenticeship model, its dimensions, and its position in clinical education.  In this scoping review study, the keywords including Cognitive Apprenticeship Model, Clinical Teaching, and Medical Education were searched in Scopus, Web of Science, EMBASE, and Medline databases between 2009 and 2021. After removing duplicates and considering the direct relationship of the articles to the study’s aim, full texts were reviewed. | A total of 170 records were found, out of which 15 articles were finally examined from three aspects including, introduction of cognitive apprenticeship model, position of cognitive apprenticeship model in clinical education, and evaluation of cognitive apprenticeship model. A cognitive apprenticeship model is a powerful tool for applying theoretical knowledge to clinical experiences and practicing skills through observation, participation, clinical reasoning, and independent clinical practice. | Developing the educational competencies of clinical faculty members and providing suitable opportunities for them to apply the dimensions of the cognitive apprenticeship model in clinical education has positive effects on the effort, perseverance, and future performance of students. | NA |
| 134 | Brooks. et al.  2009 | Medical Education and the Tyranny of Competency | Reflective | This article examines the intellectual origins and hidden assumptions of this concept and argues that it is an inadequate, and even harmful, concept to use as a guiding motif for professional education. The competency model—which tends to be top-down and prescriptive—does not provide the frame- work for objective educational assessment that it claims to provide | NA | The alternative apprenticeship model is more appropriate for professional education and is more consistent with what psychological research has shown about the acquisition of expertise. | NA |
| 135 | Stalmeijer et al,  2008 | Cognitive apprenticeship in clinical practice: can it stimulate learning in the opinion of students? | Qualitative | Learning in clinical practice can be characterised as situated learning because students learn by performing tasks and solving problems in an environment that reflects the multiple ways in which their knowledge will be put to use in their future professional practice. Collins et al. introduced cognitive apprenticeship as an instructional model for situated learning comprising six teaching methods to support learning: modelling, coaching, scaffolding, articulation, reflection and exploration. Another factor that is looked upon as conducive to learning in clinical practice is a positive learning climate. We explored students’ experiences regarding the learning climate and whether the cognitive apprenticeship model fits students’ experiences during clinical training. In focus group interviews, three groups of 6th-year medical students (N = 21) discussed vignettes rep- resenting the six teaching methods and the learning climate to explore the perceived occurrence of the teaching methods, related problems and possibilities for improvement. | The students had experienced all six teaching methods during their clerkships. Modelling, coaching, and articulation were predominant, while  scaffolding, reflection, and exploration were mainly experienced during longer clerkships and with one clinical teacher. The main problem was variability in usage of the methods, which was attributed to teachers’ lack of time and formal training. The students proposed several ways to improve the application of the teaching methods. | The results suggest that the cognitive apprenticeship model is a useful model  for teaching strategies in  undergraduate clinical training and a valuable basis for evaluation, feedback, self-assessment and faculty development of clinical teachers. | NA |
| 136 | Remmen et al., 2000 | An evaluation study of the  didactic quality of clerkships | Quantitative | Objective was “to use quantitative methods to further explore student perceptions of their clinical teaching.”  Monthly questionnaire administered to final-year medical students at the University of Antwerp, Belgium. | The response rate was 83.9%. Many of the qualitative findings were reproduced. Educational resources were not optimally used. The junior doctor was the most important clinical teacher. Many activities were passive experiences. Coaching, feedback and supervision associated most with general satisfaction of the clerkship, however, these dimensions were often considered suboptimal. | Clinical clerkships do not automatically provide an ideal learning environment for medical students. | 7 |
| 137 | Northup et al. 2013 | Procedural Competency of Gastroentrology Trainees: From Apprenticeship to  Milestones | Descriptive | Training in medicine has transformed over recent decades. With the advances in imaging, procedures, and technology, combined with the influence of third-party payers, there is more scrutiny on competency in medical training than perhaps any other time in medical history. In addition, the progressive blurring of the line between surgical and nonsurgical physicians has resulted in many invasive procedures being performed by internal medicine physicians and subspecialists. Excluding childbirth and delivery services, internists perform more invasive procedures than any of the surgical specialties in both the outpatient and inpatient realms. Despite this, internal medicine subspecialty training programs have struggled to develop standardized methods for teaching and evaluating procedural competence in trainees.  Understanding the weaknesses in the established numerical threshold system, the Accreditation Council for Graduate Medical Education (ACGME) has brought forth a new approach to evaluation of trainees and training programs referred to as the Next Accreditation System. One of the keystones of the new system is a “milestone”-based promotion and evaluation system for medical and surgical trainees. The proposed system assesses the progress of a trainee in reaching outcome-based milestones, including those for procedural skill acquisition, related to each core competency. These milestones are meant to be grounded in patient-centered outcomes that will be scaled by year of training, yet the specific milestones to be used for procedural competencies are not currently specified by the ACGME, American Board of Internal Medicine, or in the literature itself. | NA | In conclusion, the methods for assessing competency during endoscopic training in the United States are in transition. A general gestalt and threshold number of procedures will no longer be adequate for promotion and certification. The ACGME is in the process of developing a system of concrete, outcome-based milestones in training that will assess competence, meanwhile supporting the best clinical practices and quality in patient care and procedural performance. This transition is only beginning and will likely take years to fully implement, although specific milestone development is well underway. To implement the type of procedural milestone system that will improve the quality of endoscopic training in the United States, collaboration in the development, execution, data collection, and validation of these measures will be needed to enter the next era of physician training. | NA |
| 138 | Yardley et al.,  2012 | Experiential learning: AMEE guide No. 63 | Review paper | Not indicated | Not indicated | Not indicated | NA |
| 139 | Jochemsen-van der Leeuw et al., 2015 | Learning from a role model: A cascade or whirlpool effect? | Qualitative | The aims of this study were to gain insight into factors that encourage clinical trainers to incorporate what they have learned in CPD/FD into their role model function and the factors that influence conveyance from master to apprentice. We conducted a qualitative study using semi-structured interviews with GP trainers and their trainees. | Twenty-four GP trainers who  completed a CPD/FD course and  sixteen of their trainees participated in the study. Analysis of their statements enabled the identification of factors that affect the amplification of the competences of clinical trainers and their awareness of being a role model, the applicability in training practice and conveyance to the trainee. | As a result of interactions between the trainer, trainee and patient, it seems more accurate to represent the conveyance of competences from master to apprentice using the image of a whirlpool rather than a cascade, with the influential factors and interactions functioning as filters, causing a decline in the effectiveness of CPD/FD. Using the filters as a basis for turning-points for improvements around the whirlpool could increase the effectiveness of CPD/FD. | COREQ:  482-488 |
| 140 | Merritt et al. 2017 | Apprenticeship to  Entrustment: A Model for Clinical Education | Review Paper | Not indicated | Not indicated | Not indicated | NA |
| 141 | Symmes et. al. (2024) | Fostering apprenticeship in hospital medicine education: Establishing a taxonomy for direct care hospitalist teaching services | Qualitative | Proposed the nomenclature of direct care hospitalist teaching services (DCHTS). Direct care highlights the fact that the attending hospitalist is providing direct clinical care to some or all of the patients as the responding clinician, hospitalist emphasizes our role in the medical system, and teaching service solidifies our professional identity as educators. | NA | NA | NA |
| 142 | Archibald, D et. al. (2021) | Capturing the impact of cultural differences in residency | Qualitative | To assess the extent cultural differences create barriers for residents, particularly but not exclusively for international medical graduates (IMGs).  In 2014–15 a questionnaire was developed with subscales assessing areas such as sense of hierarchy, individuality versus teamwork, and risk tolerance. We refined the instrument by subjecting it to a review panel of experts in postgraduate education followed by “think aloud” sessions with residents. | The Impact of Cultural Differences on Residency Experiences (ICDRE) questionnaire revealed a few items for which the Canadian Medical Graduates and International Medical Graduates differed in their mean opinion. The groups were not substantially different overall, but we did observe an interesting diversity of cultural beliefs within each group. | We suggest that the ICDRE may be useful in identifying beliefs which may present challenges to an individual resident or in capturing trends in a resident population so that a specialty program can address the trends proactively. The instrument also provides language with which to anchor preceptors’ evaluations of residents’ professionalism and may serve as an interventional coaching tool. | NA |
| 143 | Torralba et al.,  2020 | Psychological safety, the hidden curriculum, and ambiguity in medicine | Case based review | Discusses the need for psychological safety to foster learning and facilitate high-performing teams in the clinical learning environment | Not indicated | Psychological safety improves communication and teamwork by allowing individuals to be comfortable expressing and being themselves, as well as comfortable sharing concerns and mistakes without fear of embarrassment, shame, ridicule, or retribution.  Commonly studied in the context of patient safety and quality improvement, psychological safety should extend towards medical education particularly in the context of allowing medical students and postgraduate trainees to be able to voice clinical reasoning in the face of ambiguity.  Educators take on a leadership role when having learners under their supervision; as leaders, educators are the prime movers of psychological safety  Learners in the process of developing their self-identity in the context of their chosen profession adopt not only knowledge and skills within the framework of an explicit and formal curriculum but also norms and values from daily behavior and language educators present in the clinical learning environment of learners; these norms and values are collectively part of the hidden curriculum. | NA |
| 144 | Sheu et al.,  2021 | How Do Clinical Electives during the Clerkship Year Influence Career  Exploration? A Qualitative  Study | Qualitative | Students in the institution of study participate in 3 2-week clinical electives called Clinical Immersive Experiences (CIExes) during their clerkship year. These CIExes were categorised into 4 different types, one of which was apprenticeship. Semi-structured interviews were done of the students. Authors coded and analyzed transcripts using template analysis. | The study mainly looked into the significance of clerkships on students’ career exploration and concluded that it is of benefit, by creating a positive learning environment that allowed deep relationships to develop and that supported a strong sense of wellbeing. | In a clerkship, students were able to more successfully develop a student-supervisor mentoring relationship that helped with learning and guided career choices. | NA |
| 145 | Johnson-Laghi et al.  2022 | Integrating Cognitive  Apprenticeship Into  Gastroenterology Clinical Training | Descriptive | Gastroenterology, like other specialties in medicine, is based on apprenticeship. Apprenticeship has long been considered the natural way of learning. A teacher demonstrates how to perform a task while the learner observes  and eventually performs the task with decreasing direction from the teacher. The learning process based on traditional apprenticeship takes a long time and can have various results. More importantly, the process of problem solving is  often invisible unless the teacher’s thinking is deliberately brought to the surface. In an effort to make such tacit knowledge explicit, Brown et al introduced the concept of cognitive apprenticeship, a “model of instruction that works to make thinking visible.” Cognitive apprenticeship has been explored in several medical and surgical domains and for gastrointestinal procedural skills training; however, there is little implementation of cognitive apprenticeship in gastroenterology clinical training.9 In this article, we discuss cognitive apprenticeship, its major strategies, and how to introduce them to gastroenterology fellowship education. | NA | Cognitive apprenticeship is a training method that prioritizes learner understanding and application of knowledge to real-life tasks. It is the practice of making the tacit knowledge of the expert “visible” to the learner. Modeling, coaching, scaffolding, articulation, self-reflection, and exploration are important teaching strategies that are used increasingly in medical and surgical education. Gastrointestinal procedure skills, outpatient clinical care, and management of acute gastrointestinal pathologies are opportune settings for the application of cognitive apprenticeship strategies. The implementation of cognitive apprenticeship in a fellowship program will require active participation from educators. The act of making tacit knowledge explicit or “visible” may prove challenging for most experienced physicians. Thus, formal training in all 6 strategies should be considered for faculty members involved in trainee education, with the goal of providing trainees with the skills needed to carry out expert problem-solving processes as independent practitioners. | NA |
| 146 | Hari et al. 2022 | Describing Ultrasound Skills Teaching by Near Peer and Faculty Tutors Using Cognitive Apprenticeship | Quantitative | Ultrasound skills are becoming increasingly important in clinical practice but are resource intensive to teach. Near-peer tutors often alleviate faculty teaching burden, but little is known about what teaching methods near-peer and faculty tutors use. Using the lens of cognitive apprenticeship, this study describes how much time faculty and near-peer tutors spend on different teaching methods during abdominal ultrasound skills training. Sixteen near-peer and 16 faculty tutors were videotaped during one 55min practical ultrasound lesson with randomly assigned students. Videos were directly coded using Cognitive Apprenticeship teaching methods and activities. Segment durations were summed up and compared quantitatively. | All 32 tutors spent most of the time on observing and helping students (Coaching, Median 29:14minutes), followed by asking open and stimulating questions (Articulation, 12:04 minutes and demonstrating and giving explanations (Modeling, 04:50 minutes). Overall, distributions of teaching methods used were similar between faculty and near-peer tutors. However, faculty tutors spent more time on helping students manually, whereas near-peer tutors spent more time on exploring students’ learning gaps and establishing a safe learning climate. Cognitive Apprenticeship was well suited as observational framework to describe ultrasound skills. | Ultrasound train-the-tutor programs should particularly focus on coaching and articulation. Near-peers’ similar use of teaching methods adds to the evidence that supports the use of near-peer teaching in ultrasound skills education. | MERSQI:86 |
| 147 | Gheasuddin et al. 2021 | Use of an apprenticeship  model to facilitate prescribing learning on clinical placements | Descriptive | Implementation of an apprenticeship model at  UCL - in which students are directly assigned to work within a specific healthcare team - during this disrupted year has meant that we as a student body are overall more involved with individual patients’ journeys. This has meant that we feel more confident not only in suggesting medications for patients but also in following up on the prescriptions ourselves. The apprenticeship model also allows immediate feedback and negates the need for a specific medical student pre-prescription as suggested in Tip 5, allowing us to simultaneously learn from our mistakes while ensuring the utmost patient safety, as our prescriptions are always checked by junior doctors. | NA | The apprenticeship model was brought in to mitigate the effects of COVID-19 disruption on our learning. However, we feel that it has been beneficial in many ways, particularly in targeting the universal lack of prescribing practice throughout medical school. We hope that other schools can take this on board when developing their curriculums. | NA |
| 148 | Lee, 2008 | Graduate Medical  Education in Ophthalmology: Moving From the Apprenticeship  Model to Competency-  Based Education | Review Paper | Historically, graduate medical education in the United States has relied heavily on the apprenticeship model (ie, “see one, do one, teach one”). In this apprenticeship model, the “apprentice” (ie, the resident or fellow) serves under the guidance of an experienced faculty master. The model unfortunately has significant  limitations that have driven calls for transformation to a more competency-based model of education. The transition will ideally better align the training program’s objective of achieving individual resident competency with the parallel but overarching goals of aggregate, programmatic, and systemwide improvements in health care. The end result of this multiyear process will hopefully allow professional educators, the public at large, third-party payers, and internal and external regulatory agencies to infer a physician’ s ability to “do” in the real world (ie, doing the right thing in the right way for the right reason and at the right time) | Not indicated | In summary, the future of ophthalmic resident and fellow education is in transition from the apprenticeship model to the competency-based model. The new tools being developed for teaching and assessing the ACGME competencies will likely need to be deployed both upstream in the educational process at the medical school level and downstream at the level of the practicing physician (ie, maintenance of certification). Ideally, the same tools developed to assess residents and fellows would be used for faculty members and practicing physicians. | NA |
